# Loss of Peroxiredoxin 6 Drives Age-Related Klf9/NF-*κ*B/Nlrp3 Inflammasome Activation and Pyroptosis: Therapeutic Rescue by Prdx6

**DOI:** 10.3390/antiox15050532

**Published:** 2026-04-23

**Authors:** Bhavana Chhunchha, Eri Kubo, Deepali Lehri, Dhirendra P. Singh

**Affiliations:** 1 Department of Ophthalmology and Visual Sciences, University of Nebraska Medical Center, Omaha, NE 68198, USA; 2 Department of Ophthalmology, University of Kanazawa, Kanazawa 9200293, Japan

**Keywords:** Prdx6, Nlrp3, Klf9, NF-*κ*B, aging, oxidative stress

## Abstract

The abnormal activation of the Nlrp3 (Nod-like receptor pyrin 3) inflammasome, in response to oxidative stress or impaired antioxidant defense, is linked to aging-related diseases. Previously, we have shown that Peroxiredoxin (Prdx)6 deficiency triggers reactive oxygen species (ROS)-dependent activation of Kruppel-like factor (Klf)9/Nlrp3 inflammasome in aging lens epithelial cells (LECs). Herein, we test the therapeutic efficacy of Prdx6 delivery in abating the oxidative stress-induced aberrant activation of the Klf9/NF-*ĸ*B/Nlrp3 pathway and subsequent pyroptotic cell death in LECs and *Prdx6*-deficient (*Prdx6^−/−^*) LECs. Similar to aged LECs, *Prdx6*-depleted LECs exhibited activation of Nlrp3 inflammasome components—including ASC, Caspase-1, IL-1β, IL-18, GSDMD—and displayed heightened sensitivity to H_2_O_2_/UVB-induced oxidative damage. The delivery of TAT-HA-Prdx6 or the overexpression of Prdx6 in *Prdx6^−/−^* mLECs significantly suppressed the aberrant activation of these inflammatory components and restored redox balance by eliminating ROS levels during oxidative stress. Similarly, TAT-HA-Prdx6 effectively internalized into SRA-hLECs and suppressed the H_2_O_2_- and/or UVB-induced upregulation of Nlrp3 and its components. Furthermore, the oxidative stress or *Prdx6* deficiency led to increased Nlrp3 promoter activity and NF-*ĸ*B activation, accompanied by decreased cytosolic I*ĸ*Bα and increased phosphorylation of I*ĸ*Bα; these alterations were reversed by Prdx6 overexpression. The elevated Klf9 transcription observed in aging and *Prdx6^−/−^* mLECs or under oxidative stress was also inhibited by Prdx6 delivery. Additionally, *Prdx6^−/−^* mLECs and aging LECs displayed increased TXNIP and reduced TRX levels, which were normalized by Prdx6 restoration. Collectively, this study provides the first evidence that the loss of Prdx6 drives aberrant activation of Klf9/NF-*ĸ*B/Nlrp3 inflammasome axis, leading to pyroptotic cell death. Prdx6 delivery represents a promising therapeutic strategy to rescue cells from pyroptosis (oxidative stress-induced inflammatory cell death).

## 1. Introduction

Aging and oxidative stress share common molecular pathways that drive age-related diseases (ARDs); treating ARDs may enhance resilience against others [1,2,3]. Excessive ROS-driven oxidative stress promotes cell death and underlies numerous pathological conditions/diseases, including neurodegenerative, cardiovascular, and ocular diseases such as age-related cataracts [4,5,6,7,8]. Cataract is the world’s leading cause of blindness [9,10]. Approximately 94 million individuals aged 50 and older worldwide have suffered from visual impairment due to cataractogenesis. This number is expected to increase as the population ages, resulting in a further rise in the number of cataract patients [10,11,12]. In the U.S., more than 100,000 cataract surgeries are performed yearly, at a cost of approximately USD 4 billion annually [13,14]. It is noteworthy that if cataract could be delayed by 10 years, this economic burden might be reduced by 45% (Vision Research: A national Plan, 1983–1987, NIH Publication No. 83-2473, Vol. 2, Part 3 [15]). Although eye care services (by surgery)/intraocular lens (IOL) implantation successfully reduced cataract burden, they could not meet the rising requirement due to the growing aging population, specifically in developing countries, including post-surgical complications [16,17,18]. Thus, there are pressing biomedical and social challenges to identify factor(s) responsible for ARC and to develop therapies based upon those factor(s). To understand oxidative/aging-pathobiology, using the eye lens, one of the best model systems to delineate the oxidative/aging-driven pathobiology, we designed this study to identify factor(s) responsible for the pathobiology of the eye lens, and thereby develop therapeutic molecule(s). Working on this research line, other investigators and our group have shown that ROS can promote survival signaling at physiological levels but drive damage when elevated [19,20,21]. In basal conditions, ROS are tightly regulated by antioxidants, such as Prdx6, which is essential for signaling and survival [19,20]. Prdx6, traditionally known for its peroxidase role in maintaining H_2_O_2_ homeostasis, is inactivated by hyperoxidation, leading to H_2_O_2_ accumulation and stress signaling [20,22,23,24,25,26,27].

Peroxiredoxins (Prdxs) are small antioxidant proteins having six mammalian isoforms (Prdx1–6) that reduce peroxides via redox-active cysteines and are classified as typical and atypical 2-Cys (Prdx1–5) and 1-Cys (Prdx6) [28,29,30,31,32,33,34,35,36,37,38]. Prdx6 uniquely contains only one conserved cysteine (Cys47) and uses glutathione (GSH) or DTT as electron donors. Trifunctional Prdx6 processes GSH peroxidase, Ca^2+^-independent phospholipase A2 (aiPLA_2_), and lysophosphatidylcholine acyltransferase (LPCAT) activities [28,29,33,39,40,41,42,43]. Moreover, as a non-selenoprotein, the role of Prdx6 has recently been shown to have synergistic interactions with melanoproteins like GPx4 [44,45,46]; therefore, it is involved in selenium metabolism and contributes to the expression and activity of selenoproteins. Prdx6 does so by facilitating selenium availability, which is a prerequisite for its expression and activity [47,48,49]. Prdx6 is widely expressed in tissues, including the eye, brain, lungs, liver, and GI tract [28,39,40,42,43,50]. It is enriched in epithelial cells and localized to cytosol, mitochondria, endoplasmic reticulum (ER), lysosomes, and plasma membranes, underscoring its central role in oxidative defense and cell survival across organ systems [51,52,53,54,55,56,57,58,59,60,61]. Nevertheless, several pieces of evidence reveal that Prdx6 occupies a vital position in the regulation of redox-signaling, from cell survival to cell health, as its deficiency leads to oxidative stress-related aging disorders. Age-dependent decline or functional impairment of Prdx6, and, thereby, the increase in oxidative stress, has been found to be related to different types of cell death, like pyroptosis, apoptosis and ferroptosis, leading to cataract formation [8,22,50,58,60,62,63,64,65,66,67,68,69]. These studies indicate that Prdx6 is an essential molecule to maintain cell health and cellular protection against oxidative stress and age-associated oxidative stress-induced pathobiology of the lens [70].

Nlrp3 inflammasome is the most extensively studied inflammasome. It has been demonstrated that Nlrp3 inflammasome activation is a common disease pathway that, once activated, can amplify and sustain inflammation [71]. Recently, several studies have demonstrated direct links between oxidative/aging-evoked activation of the Nlrp3 inflammasome inflammatory pathway and the onset of cataractogenesis [72,73,74,75,76,77]. This includes many activation and inflammatory components that drive the maturation and release of mature interleukin (IL)-1β and IL-18, and induce pyroptosis, playing a critical role in diverse physiological and pathological processes [78]. Inflammation and oxidative stress are interrelated processes that regulate each other and contribute to the pathogenesis of numerous diseases. Evidence indicates that excessive reactive oxygen species (ROS) generation and dysregulated inflammatory responses are strongly associated with metabolic disorders [79]. ROS are key mediators of Nlrp3 inflammasome activation. The inflammasome and pyroptosis have emerged as critical mediators of inflammatory diseases; studies suggest that reducing ROS production—either directly or via activation of antioxidant pathways—can suppress inflammasome activation and pyroptosis, thereby preventing chronic tissue inflammation, oxidative stress, and organ damage [80,81]. Nlrp3 inflammasome-mediated inflammation recruits macrophages and neutrophils, which in turn enhance ROS production, establishing a feedback loop between ROS and Nlrp3 inflammasome activation [82]. ROS are upstream inducers of the inflammasomes [83].

Moreover, transcription factor NF-*ĸ*B plays a central role in cell growth and survival as well as development and proliferation, and is also involved in many pathological conditions, including pyroptosis. NF-*ĸ*B is a key regulator of immune responses, driving pro-inflammatory gene expression, inflammasome activity, and immune cell function [84]. ROS act as key signaling mediators; NF-*ĸ*B-regulated genes help to control intracellular ROS levels, while ROS can either stimulate or inhibit NF-*ĸ*B activity [84]. Dysregulated NF-*ĸ*B activation is implicated in numerous inflammatory diseases [85]. It drives the expression of pro-inflammatory cytokines and chemokines, influences inflammasome activity, and controls the survival, activation, and differentiation of innate immune cells and inflammatory cells. Activation of the Nlrp3 inflammasome in macrophages requires a priming signal in addition to specific activators [86]. Priming stimuli, such as TLRs, NLRs, or cytokine receptors, trigger NF-*ĸ*B activation, leading to the increased expression of Nlrp3 and pro-IL-1β. In resting macrophages, NF-*ĸ*B activity is low, pro-IL-1β is absent, and Nlrp3 activation cannot occur, while the expression of pro-Caspase-1, pro-IL-18, and ASC remains unaffected. Caspases interact with the IKK complex to enhance NF-*ĸ*B transcription activity and nuclear translocation [87,88]. Nlrp3 inflammasome expression is driven by NF-*ĸ*B in cultured hepatocytes. NF-*ĸ*B signaling is a prerequisite for Nlrp3 inflammasome activation in primary hepatocytes. Nlrp3 promoter contains a putative NF-*ĸ*B binding site motif, rendering LPS sensitivity in the reporter gene assay [89]. Kruppel-like factors (KLFs) physiologically regulate the NF-*ĸ*B pathway. Specifically, Klf9 inhibits breast cancer (BC) development by interacting with HDAC1 and NF-*ĸ*B subunits p50/p65, thereby suppressing the promoter activity of matrix metalloproteinase 9 (MMP9) and NF-*ĸ*B target gene, reducing its expression [90,91].

It is noteworthy that Klf9 expression was upregulated in aging LECs/LECs facing oxidative stress, which is directly associated with the reduced expression of antioxidant genes, including Prdx6 [19,20]. Klf9 expression was upregulated by various kinds of oxidative stress, such as H_2_O_2_ and paraquat [21]. Cells overexpressed with Klf9 were more susceptible to oxidative stress, while *Klf9*-deficient cells displayed resistance against oxidative stress-induced cell damage [19,20,21]. Recent reports showed that Klf9 mediated Nlrp3 inflammasome and ROS to mediate pyroptosis in trophoblasts. LPS + ATP stimulates the Klf9 expression in the preeclampsia (PE) cell model. Inhibiting Klf9 expression decreased protein expression of Nlrp3, GSDMD-N, cleaved Caspase-1, cleaved IL-1β, and suppressed the cell death induced by LPS + ATP in HTR-8/SVneo cells [92]. Chronic obstructive pulmonary disease (COPD) is marked by airway inflammation. In COPD mouse lungs, Klf9 was elevated; its inhibition reduced inflammatory cell infiltration and Nlrp3 expression. Klf9 promoted Nlrp3 inflammasome activation and inflammation by binding the miR-494-3p promoter; it repressed PTEN expression, thereby facilitating Nlrp3 inflammasome-mediated Nlrp3 inflammasome and inflammation [93].

TXNIP is a redox-sensitive component; it has been shown that ROS causes TXNIP-mediated Nlrp3 inflammasome activation [83]. TXNIP directly interacts with Nlrp3 after oxidative stress, which is critical for the activation of the inflammasome [94,95,96,97]. TXNIP binds to TRX, which inhibits the reducing activity of TRX in normal conditions [98,99,100,101,102,103]. TRX is an antioxidant enzyme that helps to protect cells from oxidative damage by reducing disulfide bonds in proteins. During oxidative stress conditions, the TXNIP–TRX complex dissociates; TXNIP can directly interact with Nlrp3, facilitating the activation of inflammasome pathways by increasing the TXNIP–Nlrp3 association [83,104,105,106]. TXNIP strongly responds to glucose and works as a central mediator of glucotoxicity in pancreatic β cells. High glucose stimulates the interaction of TXNIP with the inflammasome protein to increase IL-1β secretion by pancreatic β cells [107]. TXNIP, a regulator of insulin resistance, activates the Nlrp3 inflammasome by dissociating from TRX in an ROS-dependent manner and binding Nlrp3. Loss of TXNIP or Nlrp3 reduces IL-1β secretion and improves glucose tolerance, linking TXNIP–Nlrp3 signaling to type 2 diabetes pathogenesis [108].

Herein, we test the hypothesis that aging-associated oxidative stress or oxidative stress is involved in Nlrp3-inflammasome-mediated inflammatory cell death and pyroptosis, which is exaggerated with advancing age due to the decline in antioxidant protein Prdx6. Prdx6 deficiency in aging LECs/LECs facing oxidative stress causes increased ROS accumulation, which is directly associated with Nlrp3 inflammasome activation. In LECs with under- and overexpression of Prdx6, in the presence or absence of oxidants, herein, we demonstrate that normal cellular processes are adversely affected by the activation of Nlrp3 inflammasome signaling pathways evoked by aging/oxidative stress-generated ROS. In addition, we show that Prdx6 delivery reversed adverse aging and oxidative stress-mediated inflammatory signaling.

## 2. Materials and Methods

### 2.1. Cell Culture

SRA-hLECs: Human lens epithelial cells (hLECs) cell line (SRA01/04), immortalized with SV40, were originally derived from infant lenses during surgery for retinopathy of prematurity [109] and kindly gifted by the late Dr. Venkat N. Reddy (Eye Research Institute, Oakland University, Rochester, MI, USA). These cells were cultured in Dulbecco’s Modified Eagle Medium (DMEM, Invitrogen, Waltham, MA, USA) with 15–20% fetal bovine serum (FBS, SeraPrime, Fort Collins, CO, USA), 100 µg/mL penicillin and 100 µg/mL streptomycin in 5% CO_2_ environment at 37 °C, as described previously [62,110].

mLECs: Three types of mouse lens epithelial cells (mLECs) were used: (i) a cell line of wild-type (Prdx6^+/+^) mLECs; (ii) Prdx6-targeted mutants (*Prdx6*^−/−^) mLECs; and (iii) primary mLECs isolated from C57BL/6 mice. All animal procedures complied with the ARVO (Association for Research in Vision and Ophthalmology) “Statement for the Use of Animals in Ophthalmic and Visual Research” and were approved by the Institutional Animal Care and Use Committee (IACUC), University of Nebraska Medical Center (UNMC), Omaha, NE. All animals were maintained under specific pathogen-free conditions in an animal facility.

LECs isolated from Prdx6-targeted mutants (*Prdx6*^−/−^) and wild-type (Prdx6^+/+^) mice were generated and cultured in Dulbecco’s Modified Eagle’s Medium (DMEM; Invitrogen, Carlsbad, CA, USA) supplemented with 10% fetal bovine serum (FBS, SeraPrime, Fort Collins, CO, USA), following our published protocol [51]. *Prdx6^−/−^* mutant mice, with a fully inbred C57BL/6 background and wild-type mice of the same sex and age (Prdx6^+/+^) were used in this study. LECs were isolated from mice of identical age; Western analysis was carried out to confirm the presence of αA-crystalline [51,109], a specific marker of LECs that minimizes the variation due to genetic background. Cells from 3–5 passages were used for the experiments.

Primary mouse lens epithelial cells isolation: Primary mLECs were isolated from C57BL/6 mice of variable ages (Charles River Laboratories, Wilmington, MA, USA). Mice were maintained at 22 ± 2 °C and 55 ± 5% humidity and euthanized by cervical dislocation. Briefly, lenses were extracted and capsules were trimmed and explanted into collagen IV-coated 35 mm culture dishes containing DMEM with 15–20% FBS, with slight modifications of established protocols [51,111,112]. Capsules were spread using forceps, with cell layers oriented upward on the surface of plastic Petri dishes and allowed to attach and grow. Culture explant was trypsinized and re-cultured. Cell cultures reaching 90–100% confluency were used for this study [20,22,51]. αA-crystallin, a specific marker of LEC expression, was confirmed by Western analysis to validate LEC identity [64].

### 2.2. Lentiviral (LV) Infection

CopGFP (green fluorescence protein) control lentiviral particles (LV ShControl, sc-108084), Prdx6/GFP ShRNA (LV *Sh*-Prdx6 (human), sc-62896-VS, and LV GFP-*ShPrdx6* (mouse), sc-62897-VS) were purchased from Santa Cruz Biotechnology (Dallas, TX, USA). Lentiviral infections were performed in SRA-hLECs and mLECs, following the company’s protocol and our previously published protocol [19,20]. Briefly, SRA-hLECs and mLECs were cultured in a 6-well plate in complete medium; 24 h later, cells were washed and 2 mL fresh medium containing polybrene (5 µg/mL; sc-134220, Santa Cruz Biotechnology) was added to enhance transduction efficiency. LV GFP-ShControl and LV GFP-*ShPrdx6* lentiviral particles were added, gently mixed, and incubated overnight; 24 h later, cells were washed, and fresh complete media were added. Infected SRA-hLECs and mLECs were subcultured and treated with puromycin dihydrochloride (sc-108071, Santa Cruz Biotechnology) to select for stable integrants. Puromycin-resistant cells were maintained as stable lines and used for the current study.

For Prdx6 overexpression, Prdx6 (NM_004905) human tagged ORF clone lentiviral particles linked to pLenti-C-mGFP-P2A-Puro vector (LV-GFP-Prdx6, RC207780L4V) and corresponding lentiviral Control particles (LV-GFP-Control, PS100093V) were obtained from OriGene Technologies, Inc. (Rockville, MD, USA). The lentiviral transduction in *Prdx6^−/−^* was carried out according to the manufacturer’s instructions. In brief, *Prdx6^−/−^* mLECs were seeded in 35 mm plates in complete medium. After 24 h, the cells were washed and incubated with 2 mL of fresh medium containing polybrene (8 µg/mL; sc-134220, Santa Cruz Biotechnology) along with either LV GFP-Control or LV GFP-Prdx6 particles. Following overnight incubation, cells were washed and supplied with fresh medium. Infected *Prdx6^−/−^* mLECs were subsequently selected with puromycin dihydrochloride (sc-108071, Santa Cruz Biotechnology) to establish stable cell lines, which were then used for the experiments described in this study.

### 2.3. Protein Isolation and Western Blotting

Total protein lysate from LECs was extracted using ice-cold radioimmunoprecipitation assay (RIPA) buffer; Western analysis was performed, as described previously [20,22,51,62,113]. The membranes were probed with anti-Prdx6 and anti-TRX1 (LF-PA0187 and LF-PA0011, Ab Frontier, Seoul, Republic of Korea), Nlrp3 (#PA5-79740, ThermoFisher Scientific, Waltham, MA, USA). Caspase-1 (#24232 and #3866), ASC (#13833 and #67824), IL-1β (#122452), IL-18 (#57058 and #54943), Gasdermin D (#39754S), TXNIP (#14715) were purchased from Cell Signaling Technology (Danvers, MA, USA). NF-*ĸ*B (sc-8008), pI*ĸ*B (sc-8404) and I*ĸ*B (sc-1643) were purchased from Santa Cruz Biotechnology (Dallas, TX, USA). β-actin (A2066, Sigma-Aldrich, St. Loius, MO, USA)/Tubulin (ab44928, Abcam, Waltham, MA, USA) were used as an internal control to monitor protein expressions. After washing, membranes were incubated with appropriate HRP-conjugated secondary antibodies (sc-2354 and sc-2768, Santa Cruz Biotechnology). Specific gene protein bands were visualized using enhanced chemiluminescence Western Blotting Luminol Reagent (sc-2048; Santa Cruz Biotechnology) and imaged with a FUJIFILM-LAS-4000 luminescent image analyzer (FUJIFILM Medical Systems Inc., Hanover Park, IL, USA).

### 2.4. RNA Isolation and Quantitative mRNA Analysis by RT-qPCR

Total RNA was extracted from human and mouse LECs using TRIzol reagent (Invitrogen, Waltham, MA, USA) based on the single-step guanidine thiocyanate/phenol/chloroform extraction method (Trizol Reagent, Invitrogen). A total RNA (0.5 to 5 µg) was reverse-transcribed into cDNA using Superscript II RNAase H reverse-transcriptase (Invitrogen). Gene expression levels of Prdx6, Nlrp3, ASC, Caspase-1, IL-1β, IL-18, GSDMD, NF-*ĸ*B, TXNIP, TRX and β-actin were quantified using real-time PCR (RT-qPCR) with SYBR Green Master Mix (Roche Diagnostic Corporation, Indianapolis, IN, USA) on a Roche^®^ LC480 Sequence detector system (Roche Diagnostic Corporation). PCR amplification was performed with an initial hot start at 95 °C for 5–10 min, followed by 45–55 cycles of 10 s at 95 °C, 30 s at 60 °C, and 10 s at 72 °C. Primer sequences used for RT-qPCR are shown below, in Table 1.

### 2.5. Cell Viability Assay

Cell viability was measured using the MTS assay to evaluate the effects of H_2_O_2_ and/or UVB on LECs. Toxicity was analyzed using the MTS assay. The assay was performed using the CellTiter 96^®^ Aqueous One Solution Proliferation Kit (Promega, Madison, WI, USA) according to the manufacturer’s method and as described previously [51,113,114]. Colorimetric assay measures the reduction of the MTS tetrazolium compound to a soluble formazan product by metabolically active cells. After treatment, MTS reagent was added directly to the culture wells; cells were incubated for 2 h. Absorbance OD was read at 490 nm using a microplate reader (DTX 880, Multimode detector, Molecular Device, San Jose, CA, USA). The data was normalized with the absorbance values of the untreated control(s).

### 2.6. Quantitation of Intracellular ROS Level by CellROX^®^ Deep Red Reagent

ROS intensity was measured using CellROX^®^ Deep Red Oxidative Stress Reagent (Catalog No. C10422, ThermoFisher Scientific) following the company’s protocol [20,22,113]. Briefly, LV ShControl and LV *ShPrdx6* infected SRA-hLECs (5 × 10^3^) and mLECs were cultured in a 96-well plate; 24 h later, cells were exposed to different concentrations of H_2_O_2_ and/or UVB, as indicated in the figure, for 6 h, following incubation with 5 µM of CellROX deep red reagent for 30 min at 37 °C. A total of 3.7% formaldehyde was used to fix the cells for 15 min. Fluorescence intensity was measured at excitation 640 nm and emission 665 nm using Spectra Max Gemini EM (Molecular Devices, San Jose, CA, USA).

### 2.7. ROS Level by H_2_-DCF-DA in LECs

Intracellular ROS levels were measured using the fluorescent dye 2′,7′-dichlorofluorescin diacetate (H_2_-DCF-DA), a cell-permeable, nonpolar compound that is enzymatically deacetylated and oxidized into the fluorescent form, dichlorofluorescein (DCF) [20,22,62,113]. Prdx6^+/+^ and *Prdx6^−/−^* mLECs were trypsinized and seeded in 96-well plates and 12–14 h later, cells were washed and transduced with TAT-HA-Prdx6 in DMEM media containing 0.2% BSA for 3 h and then exposed to H_2_O_2_ and/or UVB, as indicated in the figure legends. After 6 h of H_2_O_2_ or UVB exposure, media were replaced with Hank’s Balanced Salt Solution (HBSS) containing H_2_-DCF-DA (10 µM) dye. Cells were incubated for 30 min; intracellular fluorescence was detected at excitation (Ex) at 485 nm and emission (Em) at 530 nm using a microplate reader (Molecular Devices, San Jose, CA, USA).

### 2.8. Caspase-1 ELISA Assay

The level of Caspase-1 in LECs was quantified using Caspase-1 ELISA kits (Human: Catalog # E4588-100; Mouse: Catalog # E4180-100; BioVision, Milpitas, CA, USA), following the manufacturer’s instructions and our published protocol [60,113]. The standard curve was generated using a series of 2-fold serial dilutions from the highest concentrations of the standards [60,113]. LECs were cultured in serum-free media for 48 h. Cell culture media were removed and the cells were collected after washing twice with 1X PBS. A cellular extract was then prepared using RIPA buffer. Before the assay, all reagents, standards, and samples were equilibrated to room temperature for 30 min. Plates were pre-washed with 1X wash solution, followed by the addition of 100 µL of standards or samples to the well and incubated for 90 min at 37 °C. After removing the standards or samples, 100 µL of Biotin-conjugated detection antibody was added following 60 min of incubation at 37 °C. Wells were washed three times with 1X wash solution before the addition of 100 µL of streptavidin-HRP (SABC) working solution and incubated for 30 min at 37 °C. Wells were washed five times with 1X wash solution and TMB substrate (90 µL) was added to each well and incubated for 15–30 min at 37 °C, protected from light. Upon color development (blue), stop solution (50 µL) was added to each well. Optical density (O.D) was immediately measured at 450 nm using DTX880 multimode detector (Molecular Devices, San Jose, CA, USA).

### 2.9. IL-1β ELISA Assay

Levels of interleukin-1 (IL-1β) were determined using commercially available ELISA kits (Human; Catalog # ab214025, Abcam, USA) and (Mouse; Catalog # ab197742, Abcam, USA), following the manufacturer’s instructions and our published protocol [60,113]. To generate the standard curve, eight serial dilutions were prepared from the stock standard. The cell culture supernatant was collected and centrifuged at 5000 rpm for 20 min; clear supernatants were aliquoted and stored at −80 °C. Briefly, all solutions were brought to room temperature before the assay. A total of 50 µL of standard and/or sample was added to each well, followed by the antibody cocktail (50 µL). Plates were incubated for 1 h at room temperature on a plate shaker. Wells were washed three times with 1X wash buffer PT; then, 100 µL of TMB Development Solution was added to each well and incubated for 10–15 min in the dark on a plate shaker set to 400 rpm. The reaction was stopped by adding 100 µL of stop solution to each well, followed by mixing on the shaker plate for one minute. The optical density (OD) was examined at 450 nm using DTX880 multimode detector (Molecular Devices, San Jose, CA, USA).

### 2.10. IL-18 ELISA Assay

IL-18 protein levels were measured in LECs using commercially available ELISA kits: (Human; Catalog # ab215539 and mouse; Catalog # ab216165, Abcam, USA), following the company’s protocol and our published protocol [60,113]. Serial dilutions were prepared from this stock standard solution to generate a standard curve. For sample preparation, cells were cultured, and supernatants were collected at 48 h. Clarified supernatants were transferred to clean tubes after centrifugation for 20 min and immediately stored at −80 °C. All solutions were brought to room temperature before use. Standard or samples (50 µL) were added to the assay plate wells, followed by the addition of 50 µL antibody cocktail. Plate wells were sealed and incubated on a plate shaker at 400 rpm for 1 h at room temperature. Wells were washed three times with 1X wash buffer PT. Excess liquid was removed, and TMB Development Solution (100 µL) was then added. The plate wells were incubated for 10–15 min in the dark on a plate shaker set to 400 rpm. The reaction was stopped with 100 µL of stop solution, mixed for a minute, and absorbance was read at 450 nm using the DTX880 Multimode Detector (Molecular Devices, San Jose, CA, USA).

### 2.11. Expression and Purification of Recombinant Protein, TAT-HA-Prdx6

Full-length human Prdx6 cDNA was amplified from human LEC cDNA library using Prdx6 gene-specific primers: forward primer (5′-GTCGCCATGGCCGGAGGTCTGCTTC-3′ containing *NcoI* site); and reverse primer (5′-AATTGGCAGCTGACATCCTCTGGCTC-3′) [114,115,116,117]. The amplified PCR products were purified using QIAEX II Gel Extraction Kit (Cat No. 20021, Qiagen Inc., Valencia, CA, USA). The purified Prdx6 PCR product was ligated into a TA-cloning vector (Invitrogen, Carlsbad, CA, USA) and transformed into competent cells. Plasmids were isolated from selected single colonies; the verified Prdx6 cDNA fragment was subcloned into the pTAT-HA expression vector using *NcoI* and *EcoRI* restriction sites (a kind gift from Dr. S. F. Dowdy) [65,66,115,118,119]. TAT-HA-Prdx6 plasmid DNA was transformed into Escherichia coli BL21 (DE3) cells on LB plate supplemented with 100 µg/mL ampicillin. A total of 10 mL of overnight-cultured single colonies was used to inoculate 250 mL of pre-warmed LB–ampicillin medium, which was grown at 37 °C with shaking until OD_600_ reached ~0.6. Protein expression was induced with 1 mM IPTG; the culture was incubated for an additional 5 h at 37 °C with vigorous shaking. A total of 0.5 mL of the sample was collected before and after induction; the expression was visualized using SDS-PAGE gel. After 5 h of incubation, the remaining culture was centrifuged at 4000× *g* for 20 min to pellet the cells and then stored at −20 °C overnight. Recombinant proteins were purified after thawing the pellet for 15 min on ice using QIAexpress^®^ Ni-NTA Fast Start kit column (Qiagen Inc., Valencia, CA, USA), as described [51,116,117,120]. This purified protein was quantified and frozen at −80 °C, followed by lyophilization.

### 2.12. Construction of Nlrp3 Promoter and Promoter Activity

A DNA fragment containing the 5′-flanking region of the mouse Nlrp3 gene (−1866 to +166 bps) was amplified from mouse genomic DNA by using an Advantage^®^ Genomic LA Polymerase Mix (Catalog number 639152, Takara Bio USA, Inc., San Jose, CA, USA). The amplified DNA fragment was cloned into the *SacI* and *XhoI* sites of the pGL4.21 reporter vector (Promega) and/or basic pCAT–vector (Promega), generating the Nlrp3 promoter–luciferase construct (pGL4.21–Nlrp3) and/or Nlrp3 promoter–CAT construct (pCAT–mNlrp3), respectively. The recombinant plasmid was amplified in E. coli and confirmed by DNA sequencing. Primers used for isolating the genomic DNA fragment were as follows: forward primer (*SacI* site): 5′-AAAAGAGCTCCTTCAGCCTTAGCATTTG-3′; and reverse primer (*XhoI* site) 5′-AAAACTCGAGCTTGATCCAGACGTATGTCC-3′. LECs were transfected with the pCAT-Nlrp3 (−1866 to +166 bps) and/or pGL4.21–Nlrp3 (−1866 to +166 bps) plasmids, along with Renilla, pRL–TK vector (Promega). After 24 h, cells were exposed to H_2_O_2_ or lipopolysaccharide (LPS) or UVB, as shown in the figure. Luciferase activity was measured using the Dual-Glo Luc Assay System with a 96-well plate (Promega, Madison, WI, USA) and normalized with Renilla OD. CAT ELISA was performed to determine CAT activity following the manufacturer’s instructions. Absorbance was recorded at 405 nm using a DTX880 Multimode Detector (Molecular Devices, San Jose, CA, USA).

### 2.13. Extraction of Nuclear and Cytosolic Fractions

Nuclear and cytosolic extracts were prepared, as previously described [20,115,116,121]. Briefly, human and mouse LECs were cultured in 100 mm plates. Cells were gently washed with ice-cold phosphate-buffered saline (pH 7.4), harvested using a cell scraper, and collected by centrifugation in a micro-centrifuge. The cell pellet was resuspended in cytoplasmic extract buffer (10 mM HEPES (pH at 7.9), 0.1 mM EDTA, 10 mM KCl, 0.4% (*v*/*v*) Nonidet P-40, 0.5 mM phenylmethylsulfonyl fluoride (PMSF), and 1 mM DTT and Protease Inhibitor Cocktail). After incubation on ice, the suspension was centrifuged (4 °C) at 10,000 rpm for 10 min. The supernatant (cytoplasmic extract) was collected into a fresh tube. The remaining nuclear pellet was resuspended in nuclear extract buffer (20 mM HEPES (pH at 7.9), 1 mM EDTA, 10% (*v*/*v*) glycerol, 0.4 M NaCl, 1 mM DTT, 0.5 mM PMSF and Protease Inhibitor Cocktail). The suspension was incubated with continuous vortexing for 2 h at 4 °C. The lysate was centrifuged at 14,000 rpm for 15 min. The supernatant containing the nuclear extract was transferred into fresh tubes, aliquoted, and stored at −80 °C to prevent repeated freeze–thaw cycles. The concentration of the protein was quantified using Bradford reagent and the extract was used for experiments.

### 2.14. Determination of NF-κB Activation Using HIV–1 LTR–CAT Reporter Assay

To evaluate NF-*κ*B activation in LECs and/or LECs facing oxidative stress and assess the functionality of NF-*κ*B signaling, cells were transfected with HIV–1 LTR–CAT reporter construct, either wild-type or NF-*κ*B-binding site mutant (a generous gift from Dr. Carole Kretz-Remy) [64,122]. The HIV–1 long terminal repeat (LTR) promoter contains multiple transcription factors binding sites, including those for NF-*κ*B, and is known to be highly inducible, upregulated by 12 to 159-fold under various stress conditions, including oxidative stress. HLECs or mLECs were transfected with either the wild-type (pLTR–CAT) or the NF-*κ*B site mutated version (pLTR–CAT Pstl). After 24 h, cells were exposed to H_2_O_2_ or LPS or UVB, as shown in the figure. Then, 48 h later, CAT activity was measured using a CAT ELISA kit, according to the company’s protocol. Absorbance was recorded at 405 nm using a DTX880 Multimode Detector (Molecular Devices, San Jose, CA, USA).

### 2.15. Mouse Klf9 Promoter Linked to Chloramphenicol Acetyltransferase (CAT) Reporter Plasmid

The 5′-region of the mouse Klf9 gene, spanning from −5856 to +71 bp relative to the transcription start sites, was amplified from mouse genomic DNA using an Advantage^®^ Genomic PCR Kit (Cat. No. 639103 and 639104, Clontech Laboratories, Inc, Mountain View, CA, USA, 94043). The amplified DNA fragment was ligated into the basic pCAT reporter plasmid (Promega) using *MluI* and *XhoI* restriction enzyme sites to generate the Klf9 promoter–CAT reporter construct. The plasmid was amplified and verified by sequencing, as previously described [20,22]. The primers used for isolating the genomic DNA fragment were as follows: forward primer with *MluI* site: 5′-AAAAACGCGTGGTCATCGTAGGAAAGATGTGG-3′; and reverse primer containing *XhoI* site: 5′-AAAACTCGAGCCTACGAGACACTTCTTCCC-3′. LECs were transfected with pCAT–Klf9 promoter; 48 h later, CAT activity was measured using a CAT ELISA kit according to the manufacturer’s instructions and as described previously [19,20]. Absorbance was recorded at 405 nm (DTX880 Multimode Detector, Molecular Devices, San Jose, CA, USA).

### 2.16. Statistical Analysis

All quantitative data are presented as mean ± standard deviation (SD) from the number of independent experiments indicated. Statistical significance was evaluated using Student’s *t*-test and/or one-way ANOVA, as appropriate. A *p*-value of <0.05 and <0.001 was considered statistically significant.

## 3. Results

### 3.1. Prdx6-Depleted SRA-hLECs Bore Aberrant Expression of Nlrp3-Inflammasome, Bioactive Caspase-1, IL-1β, IL-18 and GSDMD and Showed More Susceptibility to H_2_O_2_- and/or UVB-Induced Cell Damage

**Figure 1:**
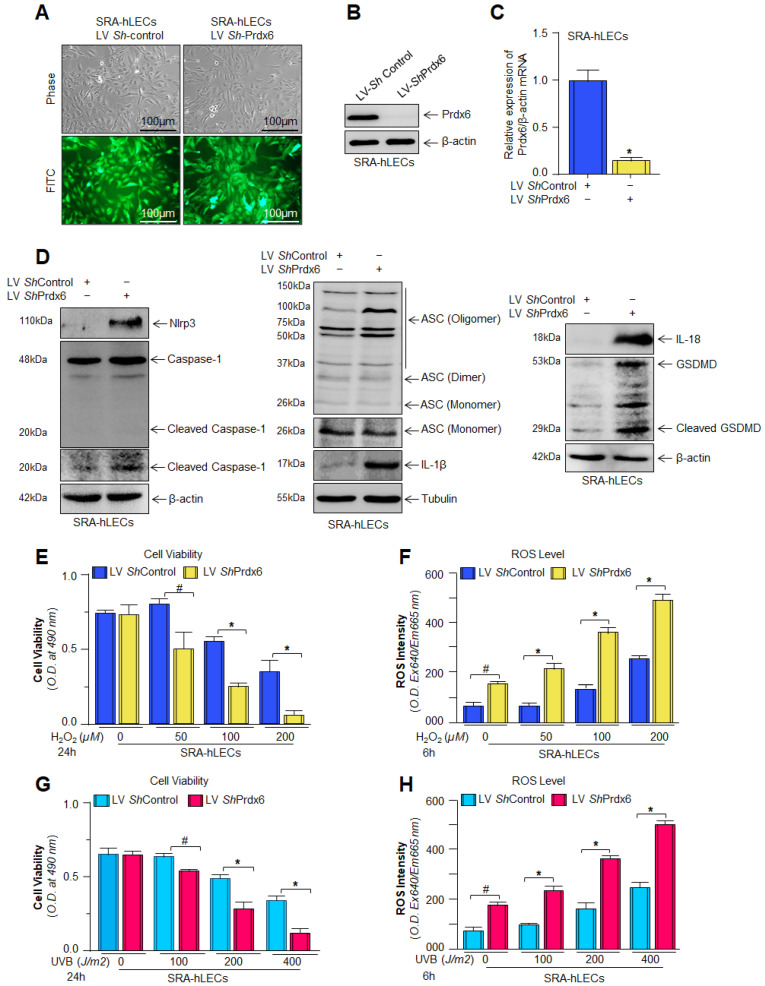
*Prdx6*-depleted SRA-hLECs were highly vulnerable to H_2_O_2_- or UVB-induced cell damage: (**A**) photomicrograph representing stably infected SRA-hLECs with LV ShControl and LV *ShPrdx6*; (**B**,**C**) expression assays showing the *Prdx6*-depleted SRA-hLECs. Total protein and RNA were isolated from stably selected SRA-hLECs infected with LV ShControl and LV *ShPrdx6*. Prdx6 protein and mRNA expression were measured using a specific probe. All histograms are presented as mean ± SD values derived from three separate experiments (*n* = 3). LV ShControl vs. LV *ShPrdx6*; * *p* < 0.001; (**D**) Prdx6 depletion enhanced the expression of Nlrp3 inflammasome and its target genes in SRA-hLECs. Total protein was isolated from LV ShControl or LV *ShPrdx6* infected SRA-hLECs and subjected to Western blot, as indicated in the figure. β-actin/tubulin was used as a loading control; (**E**–**H**) depletion of Prdx6 showed reduced cell viability and increased accumulation of ROS under oxidative stress. SRA-hLECs infected with LV ShControl and LV *ShPrdx6* were distributed into a 96-well plate, 12–14 h later, these cells were exposed to different concentrations of H_2_O_2_ or UVB. 24 h later, cell viability (**E**,**G**); and ROS levels were examined at 6 h (**F**,**H**). All histograms are presented as mean ± SD values derived from three independent experiments (*n* = 3). Statistical significance: LV ShControl vs. LV *ShPrdx6* with H_2_O_2_-treatement, and LV ShControl vs. LV *ShPrdx6* with UVB-treatment; # *p* < 0.05, * *p* < 0.001.

A moonlighting protein, Prdx6 is vitally important to maintaining redox homeostasis. We have recently shown that, during aging and oxidative stress conditions, Prdx6 expression declined with increased accumulation of ROS and activation of Nlrp3 inflammasome. To determine whether Prdx6 is essential for Nlrp3 regulation, we stably knocked down the Prdx6 gene expression in SRA-hLECs and validated it by protein and mRNA expression analyses (Figure 1A–C). As hypothesized, we observed significantly increased protein expression of Nlrp3 inflammasome and its components in *Prdx6*-depleted SRA-hLECs (Figure 1D). It has been reported that a deficiency of the antioxidant gene, *Prdx6,* makes cells more susceptible to oxidative stress [22,60,110,113]. To confirm this, we performed a functional assay using two different kinds of oxidative stress such as hydrogen peroxide (H_2_O_2_) and ultraviolet B (UVB). The cell viability assay indicated that *Prdx6*-depleted SRA-hLECs showed significantly higher vulnerability to H_2_O_2_ (E)- and UVB (G)-induced oxidative stress, resulting in reduced cell viability (Figure 1E,G) and increased accumulation of intracellular ROS generation (Figure 1F,H) in comparison to ShControl. Data indicate that the loss of Prdx6 expression compromises antioxidant defense mechanisms and thereby promotes oxidative stress-induced cytotoxicity and Nlrp3 inflammasome-mediated inflammatory cell death.

### 3.2. Prdx6-Depleted SRA-hLECs Exhibited Heightened Sensitivity to Oxidative Stress Induced by H_2_O_2_ or UVB, as Evidenced by Increased Activation of the NLRP3 Inflammasome and Its Downstream Inflammatory Signaling

**Figure 2:**
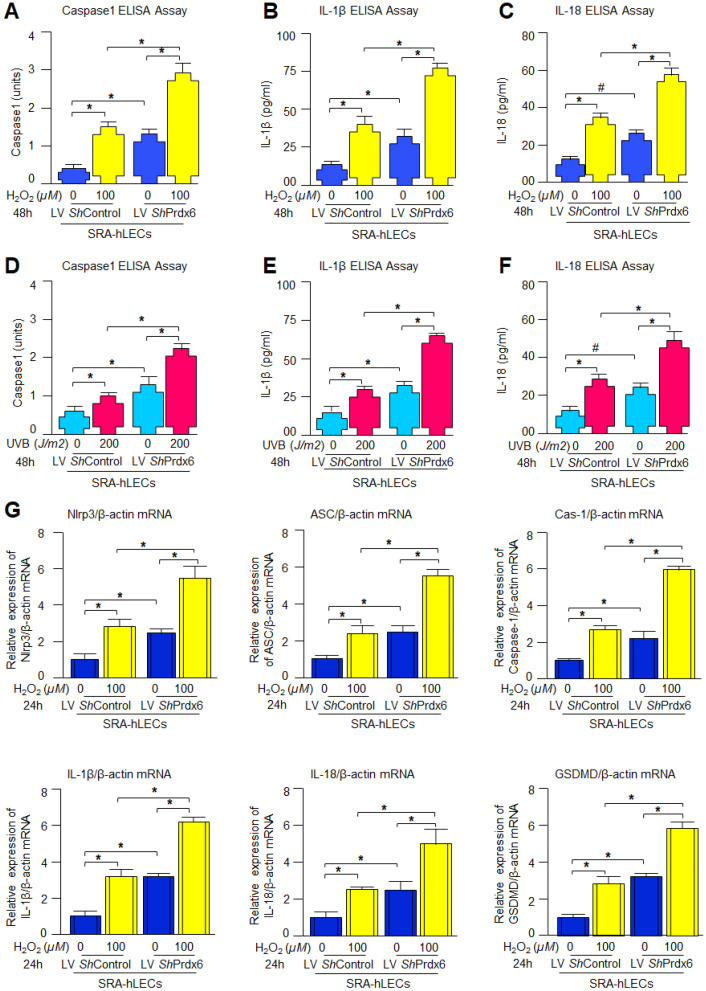
*Prdx6*-depleted SRA-hLECs showed higher sensitivity to H_2_O_2_ or UVB-induced oxidative stress and bore Nlrp3 inflammasome and its inflammatory signaling: (**A**,**D**) *Prdx6*-depleted SRA-hLECs and cells facing oxidative stress showed elevated levels of Caspase-1. LV ShControl and LV *ShPrdx6* infected SRA-hLECs were cultured and exposed to H_2_O_2_ or UVB stress, as indicated in the figure. Forty-eight hours later, a cellular extract was prepared, and an equal amount of cellular extract was used to quantify the levels of Caspase-1; (**B**,**C**,**E**,**F**) mature IL-1β and IL-18 secretion were increased in *Prdx6*-depleted SRA-hLECs and the cells facing oxidative stress. LV ShControl and LV *ShPrdx6*-infected SRA-hLECs were cultured and exposed to H_2_O_2_ or UVB stress. After 48 h, the supernatant(s) were collected and IL-1β (**B**,**E**) and IL-18 (**C**,**F**) levels were measured; and (**G**) total RNA was isolated from LV ShControl and LV *ShPrdx6* SRA-hLECs facing oxidative stress and subjected to RT-qPCR to assess the expression of Nlrp3 inflammasome and its downstream genes, mRNA, using their specific primers, as indicated. Data represent the mean ± SD values calculated from four independent experiments (*n* = 4). Significant values: 0 vs. 100 µM H_2_O_2_ in LV ShControl, 0 vs. 100 µM H_2_O_2_ in LV ShPrdx6, and LV ShControl vs. LV *ShPrdx6* treated with H_2_O_2_; 0 vs. 200 J/m^2^ UVB in LV ShControl, 0 vs. 200 J/m^2^ in LV *ShPrdx6*, and LV ShControl vs. LV *ShPrdx6* exposed to UVB; # *p* < 0.05, * *p* < 0.001 as indicated in figures.

In response to oxidative stress, Nlrp3 inflammasome-mediated activation leads to an enhancement of the level of cleaved Caspase-1, which subsequently enhances the secretion of pro-inflammatory cytokines and pyroptosis executor GSDMD. Figure 1 shows that *Prdx6*-depleted LECs enhanced Nlrp3 inflammasome-related gene expression and that these cells displayed higher sensitivity against oxidative stress. Therefore, we were interested in the status of active Caspase-1 and the active form of secreted pro-inflammatory cytokines in response to H_2_O_2_- or UVB-induced oxidative stress. We found a significant elevation of Caspase-1 level (Figure 2A,D) in *Prdx6*-depleted LECs, which were further elevated in response to oxidative stress. Next, we examined the status of pro-inflammatory cytokines in *Prdx6*-depleted SRA-hLECs and *Prdx6*-depleted SRA-hLECs facing oxidative stress in cell culture supernatant(s). Our data revealed that secretions of IL-1β (Figure 2B,E) and IL-18 (Figure 2C,F) were higher in LV *ShPrdx6*-infected SRA-hLECs compared to LV ShControl, and that the levels were markedly increased following the oxidant exposure. Furthermore, we were also interested to know the fate of Nlrp3 inflammasome and its components at the mRNA level with similar oxidative stress conditions, as mentioned above. We found that *Prdx6* depletion in SRA-hLECs significantly enhanced the expression of Nlrp3, ASC, Cas-1, IL-1β, IL-18, and GSDMD, which were further enhanced in response to H_2_O_2_-induced oxidative stress (Figure 2G), such as protein expression. Data revealed that during oxidative conditions, *Prdx6* deficiency supports the activation of Nlrp3 inflammasome-mediated inflammatory pathways.

### 3.3. Knockdown of Prdx6 in mLECs Resulted in Increased Oxidative Burden and Robust Activation of the Nlrp3 Inflammasome-Mediated Inflammatory Pathway

**Figure 3:**
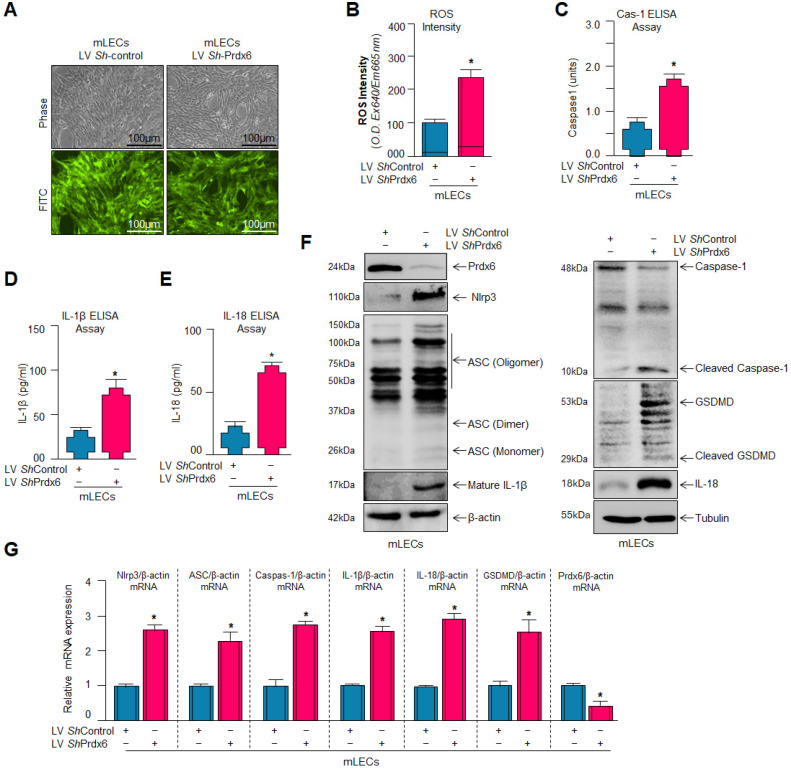
*Prdx6* knockdown in mLECs led to the increased oxidative load, which was directly linked to increased expression of Nlrp3, ASC, Caspase-1, pro-inflammatory cytokines, and GSDMD: (**A**) photomicrograph showing stably infected mLECs expressing either LV ShControl or LV *ShPrdx6*; (**B**) *Prdx6*-depleted mLECs demonstrated increased levels of ROS. ROS intensity was observed using CellROX deep red reagent. Data represent the mean ± SD values from three independent experiments (*n* = 3). LV ShControl vs. LV *ShPrdx6*; * *p* < 0.001; (**C**) *Prdx6*-depleted mLECs showed elevated levels of Caspase-1. LV ShControl and LV *Sh*Prdx6-infected mLECs were cultured, and 48 h later, cellular extract was prepared. Caspase-1 activity was measured and compared using an equal amount of cellular extract; (**D**,**E**) *Prdx6*-depleted mLECs displayed increased secretion of mature IL-1β and IL-18. LV ShControl and LV *ShPrdx6*-infected mLECs were cultured, and 48 h later, supernatant was collected to measure IL-1β (**D**) and IL-18 (**E**) levels; and (**F**,**G**) total protein and RNA were isolated from mLECs stably infected with LV ShControl and LV *ShPrdx6* and subjected to Western blot and mRNA analyses to examine the expression of Nlrp3, Caspase-1, ASC, inflammatory cytokines IL-1β and IL-18, and pyroptosis executor GSDMD, using their specific probes, as indicated. The data represent the mean ± SD values derived from four independent experiments (*n* = 4). LV ShControl vs. LV *ShPrdx6;* * *p* < 0.001.

*Prdx6^−/−^* mLECs (a model for aging) and aging LECs bear increased accumulations of ROS [22,60,62]. We also have shown in Figure 1 that *Prdx6*-deficient SRA-hLECs bore increased ROS accumulation, and these cells were more vulnerable to oxidative stress-induced insults. We were now interested to know whether mLECs, where the Prdx6 gene was knocked down, demonstrate similar results to those we obtained with the SRA-hLECs. Similar SRA-hLECs, mLECs were infected with lentiviral vectors specific to the Prdx6 gene shRNA and made a stable cell line using specific selection markers, as mentioned in Materials and Methods. *Prdx6*-depleted mLECs exhibited significantly higher ROS (Figure 3B) accumulation with increased levels of active Caspase-1 (Figure 3C), indicating plausible activation of Nlrp3 inflammasome pathway. Thus, we examined Nlrp3 inflammatory components and found that the secreted levels of mature IL-1β and IL-18 (Figure 3D,E) were significantly increased in the cell culture supernatant(s) of *Prdx6-*knockdown cells. Also, Western blot and mRNA analyses demonstrated the presence of activated Nlrp3 inflammasome with upregulated expression levels of Nlrp3, ASC, Caspase-1, IL-1β, IL-18, and GSDMD in *Prdx6*-depleted mLECs (Figure 3F,G), suggesting the correlation between inflammasome-mediated inflammatory response and loss of Prdx6.

### 3.4. Delivery of Recombinant Prdx6 (TAT-HA-Prdx6) to Prdx6-Deficient (Prdx6^−/−^) mLECs Effectively Suppressed Aberrant Nlrp3 Inflammasome Signaling

**Figure 4:**
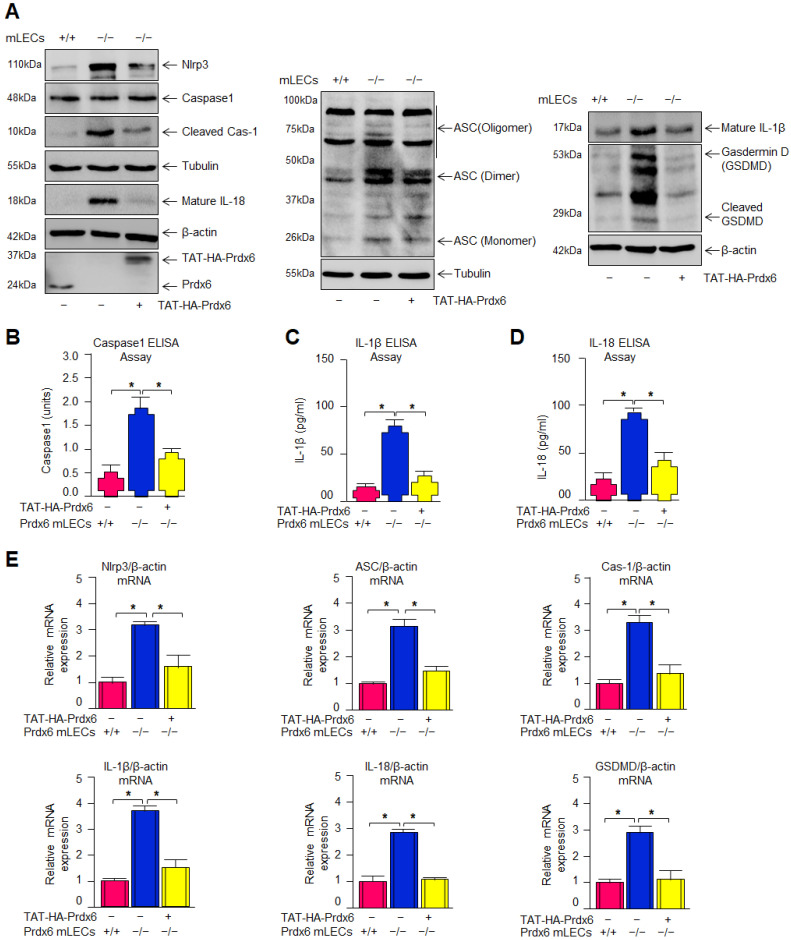
Prdx6 delivery subsided the aberrant expression and activation of Nlrp3, bioactive Caspase-1, IL-1β, IL-18, and GSDMD in *Prdx6^−/−^* mLECs: (**A**) total protein was isolated from Prdx6^+/+^ and *Prdx6^−/−^* mLECs with or without TAT-HA-Prdx6 and subjected to Western blot, as shown in the figure; (**B**) TAT-HA-Prdx6 delivery to *Prdx6^−/−^* mLECs displayed reduced levels of Caspase-1. Cellular extract was prepared and Caspase-1 status was measured; (**C**,**D**) increased secretion of bioactive IL-1β or IL-18 observed in *Prdx6^−/−^* mLECs was inhibited by Prdx6 delivery. Prdx6^+/+^ and *Prdx6^−/−^* mLECs with or without TAT-HA-Prdx6 were cultured. Forty-eight hours later, supernatant(s) were collected and measured for IL-1β (**C**) and IL-18 (**D**) levels; and (**E**) total RNA was isolated from Prdx6^+/+^ and *Prdx6^−/−^* mLECs transduced with or without TAT-HA-Prdx6 and subjected to qPCR, as indicated. The data represent the mean ± SD values of four independent replicates (*n* = 4). Prdx6^+/+^ vs. *Prdx6^−/−^* and *Prdx6^−/−^* vs. *Prdx6^−/−^* with TAT-HA-Prdx6; * *p* < 0.001.

*Prdx6* deficiency causes increased ROS accumulation and Nlrp3 inflammasome activation. Therefore, we wanted to know whether the exogenous delivery of Prdx6 inhibited the increased ROS accumulation and activated the Nlrp3 inflammasome pathway. Using almost all tools/methods noted above, the analyses of protein and mRNA indicated that the elevated expression levels of Nlrp3, ASC, cleaved Caspase-1, IL-1β, IL-18 and cleaved GSDMD found in *Prdx6^−/−^* mLECs were markedly reduced by TAT-HA-Prdx6 delivery (Figure 4A,E). Consistent with the suppression of cleaved Caspase-1 protein expression by Prdx6 delivery, the elevated levels of Caspase-1 observed in *Prdx6^−/−^* mLECs were also significantly suppressed following exogenous Prdx6 protein delivery, as demonstrated by ELISA analysis (Figure 4B). Further, we found that *Prdx6^−/−^* mLECs displayed increased levels of pro-inflammatory cytokines, IL-1β, and IL-18 at protein and mRNA expression, which could also be inhibited by the delivery of Prdx6 protein. Next, we were interested in whether Prdx6 delivery suppressed the secretion of the mature form of IL-1β and IL-18 levels. IL-1β and IL-18 ELISA assay results revealed that the Prdx6 delivery inhibited the secretion of IL-1β and IL-18 (Figure 4C,D).

### 3.5. TAT-HA-Prdx6 Delivery Mitigated Oxidative Stress and Protected the Prdx6^−/−^ mLECs Exposed to H_2_O_2_/or UVB

**Figure 5:**
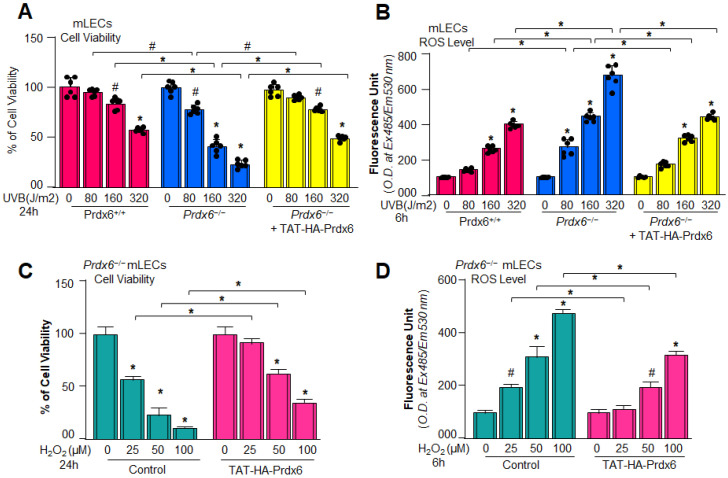
Prdx6 delivery to *Prdx6*-deficient mLECs suppressed elevated ROS levels and enhanced cell viability in response to UVB- or H_2_O_2_-induced cell death: (**A**,**C**) TAT-HA-Prdx6 protein delivery resisted against UVB- or H_2_O_2_-induced cell death in *Prdx6^−/−^* mLECs. Prdx6^+/+^ and *Prdx6^−/−^* mLECs cells were cultured in 96-well plates. *Prdx6^−/−^* mLECs were transduced with TAT-HA-Prdx6 (10 μg/mL) protein. 3 h later, these cells were exposed to different concentrations of UVB or H_2_O_2_. Twenty-four hours later, cell viability was measured, and (**B**,**D**) TAT-HA-Prdx6 protein delivery inhibited the elevated ROS level induced by UVB or H_2_O_2_ exposure in *Prdx6^−/−^* mLECs. Prdx6^+/+^ and *Prdx6^−/−^* mLECs were cultured in 96-well plates. *Prdx6^−/−^* mLECs were transduced with TAT-HA-Prdx6 (10 μg/mL) protein. After 3 h, these cells were exposed to different concentrations of UVB or H_2_O_2_ and subjected to ROS measurement at 6 h. The data reflect mean ± SD values derived from four independent experiments (*n* = 4). 0 vs. 80, 160 and 320 J/m^2^ UVB treatment, and Prdx6^+/+^ vs. *Prdx6^−/−^* and *Prdx6^−/−^* vs. *Prdx6^−/−^* supplemented with TAT-HA-Prdx6 exposed to UVB; 0 vs. 25, 50 and 100 µM H_2_O_2_ treatment, and *Prdx6^−/−^* vs. *Prdx6^−/−^* with TAT-HA-Prdx6 under H_2_O_2_ treatment; # *p* < 0.05, * *p* < 0.001.

Aging mLECs or *Prdx6^−/−^* mLECs (a model for aging) show increased accumulations of ROS levels; these LECs were more susceptible to oxidative stress [51,58,60,121]. Here, we showed that *Prdx6^−/−^* mLECs were highly susceptible to UVB-induced oxidative damage and displayed reduced viability with increased oxidative load compared to Prdx6^+/+^ mLECs (Figure 5A,B). TAT-HA-Prdx6 transduction protects the *Prdx6^−/−^* mLECs against UVB- and H_2_O_2_-induced oxidative cell death by normalizing the elevated levels of ROS accumulation (Figure 5). These findings collectively suggest that Prdx6 acts as a crucial antioxidant and anti-inflammatory regulator; its supplementation effectively restores cellular defense against oxidative damage and inflammasome activation in *Prdx6*-deficient mLECs.

### 3.6. Overexpression of Prdx6 in Prdx6^−/−^ mLECs Significantly Attenuated Nlrp3 Inflammasome Activation and Its Downstream Inflammatory Responses via ROS Suppression

**Figure 6:**
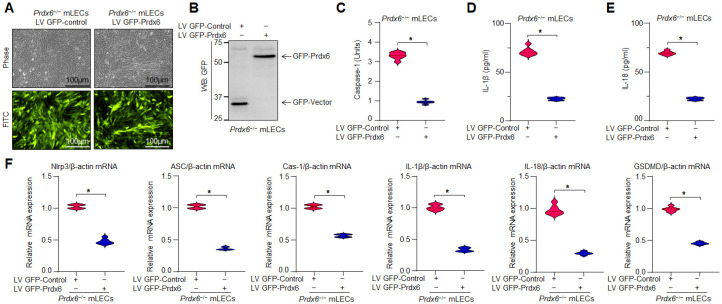
Prdx6 attenuated Nlrp3 inflammasome and its inflammatory genes ASC, Caspase-1, IL-1β, and IL-18 secretion by inhibiting ROS accumulation in *Prdx6^−/−^* mLECs: (**A**) photomicrograph representing stably infected *Prdx6^−/−^* mLECs with LV GFP-Control or GFP-tagged Prdx6 activation linked with lentiviral (LV GFP-Prdx6); (**B**) total protein was isolated from LV-GFP-Control or LV GFP-Prdx6 infected *Prdx6^−/−^* mLECs and subjected to Western blot, as indicated in the figure; (**C**) elevated levels of Caspase-1 were inhibited by Prdx6 overexpression in *Prdx6^−/−^* mLECs. LV GFP-Control and LV GFP-Prdx6-infected *Prdx6^−/−^* mLECs were cultured and 48 h later, cellular extract was prepared. Caspase-1 levels were measured using an equal amount of cellular extract; (**D**,**E**) Prdx6 overexpression normalized the increased secretion of mature IL-1β and IL-18 in *Prdx6^−/−^* mLECs. LV GFP-Control and LV GFP-Prdx6 infected *Prdx6^−/−^* mLECs were cultured, and supernatant was collected after 48 h to evaluate IL-1β (**D**) and IL-18 (**E**) levels; and (**F**) total RNA was isolated from LV GFP-Control and LV GFP-Prdx6 infected *Prdx6^−/−^* mLECs and subjected to q-PCR to assess the expression of Nlrp3, Caspase-1, ASC, cytokines, such as IL-1β and IL-18, and pyroptosis executor GSDMD mRNA expression using their specific probes, as indicated. Results represent the mean ± SD values from three independent replicates (*n* = 3). LV GFP-Control vs. LV GFP-Prdx6 in *Prdx6^−/−^* mLECs; * *p* < 0.001.

We have previously shown that the delivery of Prdx6 protects against oxidative stress-induced cell damage. The results in Figure 5 reveal that exogenous Prdx6 protein delivery inhibits Nlrp3 inflammasome activation. To validate this, *Prdx6^−/−^* mLECs were made to overexpress Prdx6 by stably infecting lentivirus, as described in Materials and Methods. Activated Caspase-1 levels, as well as elevated levels of secreted pro-inflammatory cytokines (IL-1β and IL-18) in *Prdx6^−/−^* mLECs, could be blunted by Prdx6 overexpression (Figure 6C–E). Previously, we have also shown that elevated protein expression of Nlrp3 inflammasome and its components in *Prdx6^−/−^* mLECs were normalized by Prdx6 overexpression. Similar to protein expression analysis, mRNA analysis further confirmed the suppression of Nlrp3 inflammasome gene expression (Figure 6F), demonstrating that Prdx6 restoration blunts inflammatory gene expression at transcriptional levels in *Prdx6*-deficient cells.

### 3.7. TAT-HA-Prdx6 Protein Delivery to SRA-hLECs Efficiently Suppressed UVB- or H_2_O_2_-Induced Expression of Nlrp3 Inflammasome-Associated Genes

**Figure 7:**
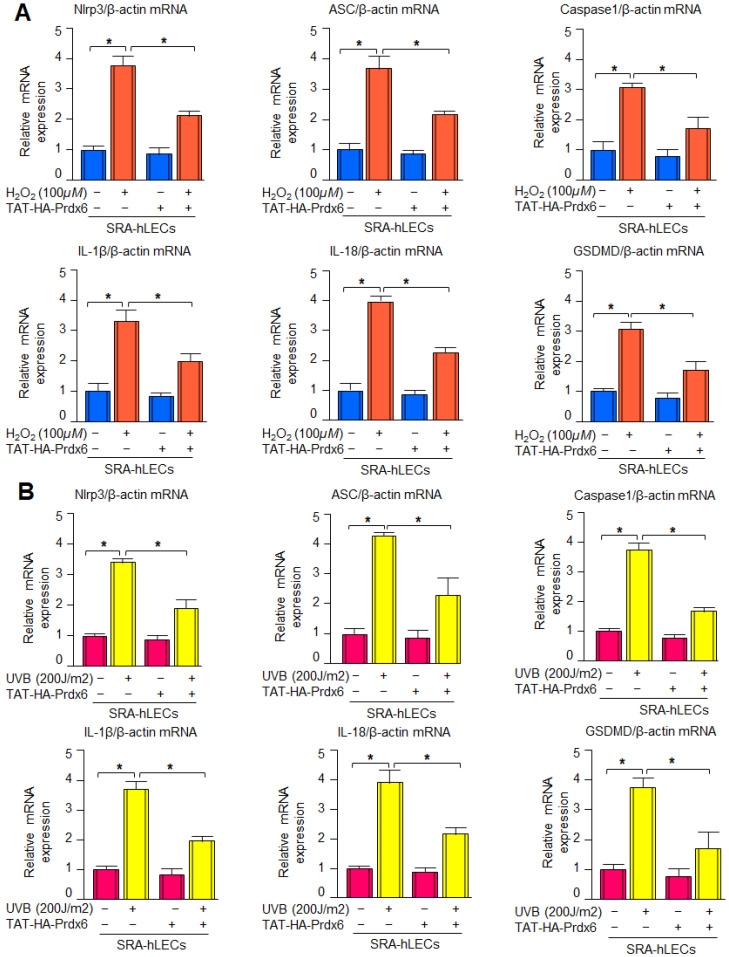
(**A**,**B**) Prdx6 delivery abated the H_2_O_2_- or UVB-driven oxidative load and increased expression of Nlrp3 inflammasome-related inflammatory components in SRA-hLECs. SRA-hLECs were pretreated with TAT-HA-Prdx6 for 3 h and followed by H_2_O_2_ (**A**) or UVB (**B**) exposure for 24 h in serum-free DMEM medium and thereafter treated for 3 days. Total RNA was isolated to assess the expression status of Nlrp3, ASC, Caspase-1, IL-1β, IL-18, and GSDMD mRNA expression using qPCR with their specific probes, as indicated in the figure. β-actin was used as a control. The data represent the mean ± SD values calculated from three independent experiments (*n* = 3). Statistical analyses: 0 vs. 100µM H_2_O_2_ treatment, and H_2_O_2_ vs. H_2_O_2_ plus TAT-HA-Prdx6; 0 vs. 200 J/m^2^ UVB treatment, and UVB vs. UVB plus TAT-HA-Prdx6; * *p* < 0.001.

Aging and oxidative stress conditions lead to the dysregulation of antioxidant defense mechanisms. We have reported that SRA-hLECs exposed to H_2_O_2_ displayed increased expression of Nlrp3, ASC, Caspase-1, pro-inflammatory cytokines and pyroptosis executor GSDMD [60]. Hence, we were interested to know whether TAT-HA-Prdx6 delivery protects the SRA-hLECs against H_2_O_2_- or UVB-induced Nlrp3 inflammasome activation. SRA-hLECs were transduced with TAT-HA-Prdx6 protein for 3 h, followed by H_2_O_2_ (Figure 7A) or UVB (Figure 7B) exposure, as indicated. Our results revealed that SRA-hLECs exposed to H_2_O_2_ or UVB displayed significantly increased expression of Nlrp3 inflammasome and its downstream signaling genes, while in cells supplemented with TAT-HA-Prdx6, the aberrant expression of the above molecules was significantly inhibited, suggesting that Prdx6 cellular abundance is critical to block oxidative stress-evoked Nlrp3 inflammasome-mediated injurious signaling, causing cellular derangements.

### 3.8. Prdx6 Negatively Regulated Nlrp3 Gene Transcription

**Figure 8:**
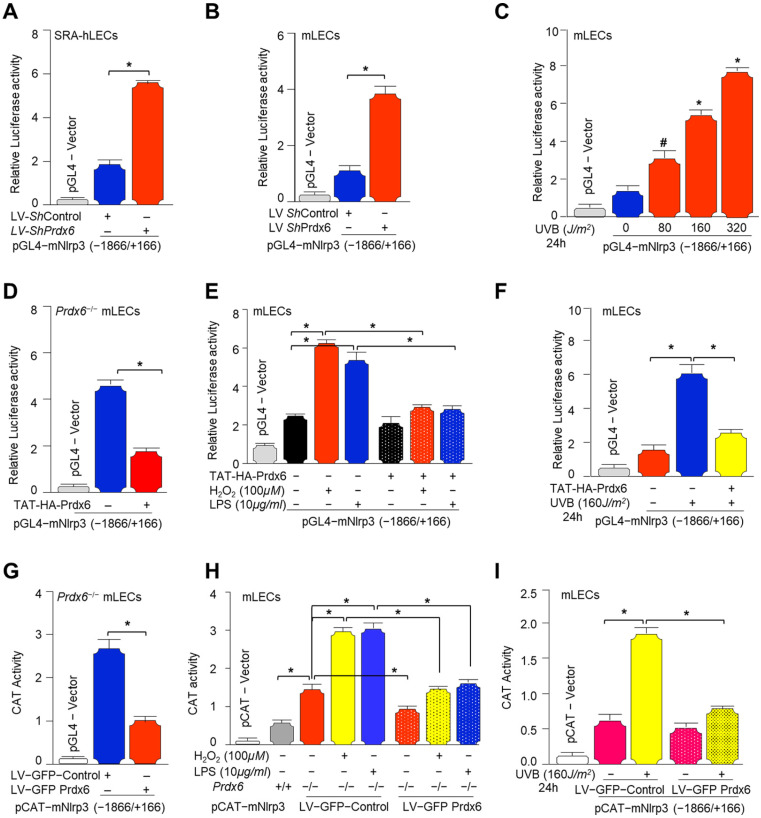
Prdx6 regulation of the transcriptional activity of Nlrp3: (**A**,**B**) *Prdx6*-depleted LECs displayed increased Nlrp3 transcription activity. Redox-active *Prdx6*-depleted SRA-hLECs (**A**) or mLECs (**B**) were transiently transfected with pGL4-mNlrp3 (−1866/+166). Forty-eight hours later, luciferase activity was measured. The data are shown as mean ± SD from three different experiments. LV ShControl vs. LV *ShPrdx6*; * *p* < 0.001; (**C**) UVB-exposed mLECs showed a significant increase in Nlrp3 transcription. mLECs were transiently transfected with pGL4-mNlrp3 (−1866/+166); 24 h later, these cells were exposed to different concentrations of UVB. Luciferase activity was measured after 24 h of UVB exposure. The results are shown as mean ± SD from four independent experiments (*n* = 4). 0 vs. 80, 160, 320 J/m^2^ UVB treatment; # *p* < 0.05, * *p* < 0.001; (**D**–**F**) Prdx6 delivery suppressed the elevated Nlrp3 transcription in redox-active *Prdx6^−/−^* mLECs and mLECs facing oxidative stress. *Prdx6^−/−^* mLECs were transiently transfected with pGL4-mNlrp3 (−1866/+166). Twenty-four hours later, these transfectants were transduced with TAT-HA-Prdx6 (10 µg/mL) for 24 h and luciferase activity was measured (**D**). Control vs. TAT-HA-Prdx6; * *p* < 0.001. mLECs were transiently transfected with pGL4-mNlrp3 (−1866/+166), and 24 h later, these cells were pretreated with TAT-HA-Prdx6 protein for 3 h, followed by H_2_O_2_ or LPS (**E**) or UVB (**F**) exposure, as indicated in the figure; then, Luciferase activity was measured after 24 h. Results reflect mean ± SD values derived from four separate experiments (*n* = 4). 0 vs. H_2_O_2_, 0 vs. LPS, H_2_O_2_ vs. H_2_O_2_ + TAT-HA-Prdx6, LPS vs. LPS + TAT-HA-Prdx6; 0 vs. UVB and UVB vs. UVB + TAT-HA-Prdx6; * *p* < 0.001; (**G**–**I**) elevated Nlrp3 transcription was inhibited by Prdx6 overexpression in *Prdx6^−/−^* mLECs and mLECs under oxidative stress conditions. *Prdx6^−/−^* mLECs and mLECs infected with LV GFP-Control and/or LV GFP-Prdx6 were transfected with pCAT-mNlrp3 (−1866/+166). 24 h later, these transfected mLECs were exposed to H_2_O_2_ or LPS (**H**) or UVB (**I**). CAT activity was measured at 48 h. *Prdx6^−/−^* mLECs (**G**), and mLECs exposed to 24 h of H_2_O_2_ or LPS (**H**), or UVB (**I**). All measurements are shown as mean ± SD from four separate experiments (*n* = 4). LV-GFP-Vector vs. LV-GFP-Prdx6, Prdx6^+/+^ vs. *Prdx6^−/−^* mLECs, 0 vs. H_2_O_2_ or LPS and/or UVB, LV-GFP-Vector (H_2_O_2_ or LPS or UVB) vs. LV-GFP-Prdx6 (H_2_O_2_ or LPS or UVB); * *p* < 0.001.

As shown in Figure 1 and Figure 2, *Prdx6* deficiency led to the activation of Nlrp3 inflammasome activity at the transcriptional level. We also have shown that Nlrp3 promoter activity was significantly higher in *Prdx6^−/−^* mLECs or mLECs facing H_2_O_2_-induced oxidative stress [60]. Similar to *Prdx6^−/−^*, we found that *Prdx6*-depleted SRA-hLECs (Figure 8A) and mLECs (Figure 8B) showed a significant increase in Nlrp3 transcription activity. Next, we intended to know whether Nlrp3 transcription is enhanced by UVB exposure, mLECs transfected with the Nlrp3 promoter, and exposed to different concentrations of UVB, as shown. Our results showed a concentration-dependent increase in Nlrp3 transcription in response to UVB exposure in mLECs (Figure 8C). Next, we also examined whether TAT-HA-Prdx6 delivery to *Prdx6^−/−^* mLECs suppresses Nlrp3 transcription. As shown in Figure 8D, the delivery of Prdx6 to the cells significantly suppressed elevated Nlrp3 transcription (Figure 8D). Similarly, pretreatment of TAT-HA-Prdx6 before H_2_O_2_, LPS, and UVB exposure reduced Nlrp3 promoter activity in wild-type mLECs, as shown in Figure 8E,F. (Note: LPS, which we have used here as a standard reagent, which has been known to activate Nlrp3 inflammasome and proinflammatory cytokines, to validate our findings). Furthermore, CAT reporter assays showed that lentiviral overexpression of Prdx6 (LV GFP-Prdx6) suppressed the enhanced Nlrp3 transcription in *Prdx6^−/−^* mLECs (Figure 8G). Furthermore, LV GFP-Control and LV GFP-Prdx6-infected *Prdx6^−/−^* mLECs were transfected with pCAT-mNlrp3 promoter following the exposure of H_2_O_2_ or LPS or UVB, as indicated. CAT activity was then measured (Figure 8H,I). Data revealed a significant reduction in Nlrp3 promoter activity in LV GFP-Prdx6-infected *Prdx6^−/−^* mLECs. Using different model systems, collectively, our results emphasize that Prdx6 inhibits oxidative stress-induced transcriptional activation of the Nlrp3 inflammasome and thereby restrains downstream inflammatory signaling.

### 3.9. Overexpression of Prdx6 Restored the Altered NF-κB Signaling in Prdx6-Deficient LECs

**Figure 9:**
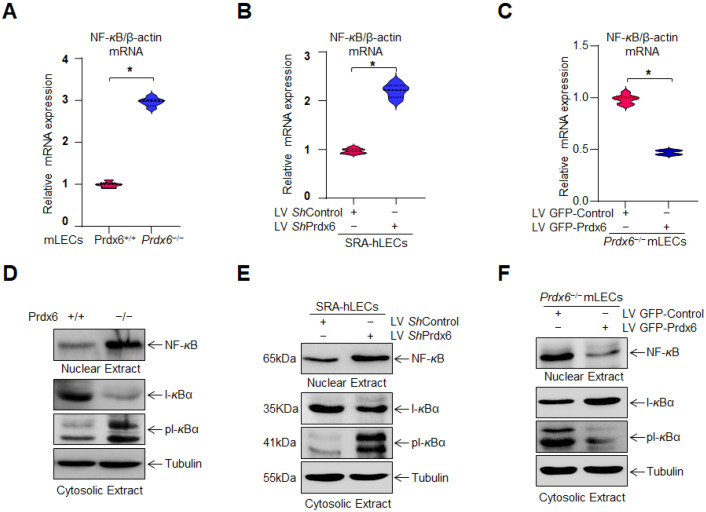
Prdx6 overexpression resulted in a reduction in NF-*ĸ*B and phosphorylated I*ĸ*Bα expression in *Prdx6*-deficient LECs: (**A**,**B**,**D**,**E**) *Prdx6^−/−^* mLECs and/or *Prdx6*-depleted SRA-hLECs showed increased expression of NF-*ĸ*B and phosphorylated I*ĸ*Bα. Total RNA and nuclear and cytosol protein extracts were isolated and subjected to RT-qPCR (**A**,**B**) and Western analysis (**D**,**E**) of NF-*ĸ*B, I*ĸ*Bα and phospho-I*ĸ*Bα with specific probes, respectively. Tubulin was used as an internal loading control. Values are expressed as mean ± SD from four separate experiments (*n* = 4). Prdx6^+/+^ vs. *Prdx6^−/−^* mLECs, LV ShControl vs. LV *ShPrdx6*; * *p* < 0.001; (**C**,**F**) increased NF-*ĸ*B and phospho-I*ĸ*Bα in *Prdx6^−/−^* mLECs were suppressed by Prdx6 overexpression. Total RNA, as well as nuclear and cytosol protein extracts, were isolated and subjected to RT-qPCR (**C**) and Western analysis (**F**) of NF-*ĸ*B and phospho-I*ĸ*Bα with specific probes, respectively. Tubulin was used as an internal control. Values are expressed as mean ± SD from four independent experiments (*n* = 4). LV GFP-control vs. LV-GFP-Prdx6; * *p* < 0.001.

NF-*ĸ*B is known to regulate the Nlrp3 inflammasome signaling pathways in different cell types. Thus, we were interested to know whether NF-*ĸ*B is involved in Nlrp3 inflammasome signaling in our model system. As shown in Figure 9A,B, NF-*ĸ*B mRNA expression was increased in *Prdx6^−/−^* mLECs and *Prdx6*-depleted SRA-hLECs. In contrast, Prdx6 overexpression reversed these effects by suppressing NF-*ĸ*B mRNA expression in *Prdx6^−/−^* mLECs (Figure 9C), suggesting the role of Prdx6 in NF-*ĸ*B transcriptional repression. However, the results revealed that Prdx6 negatively regulates NF-*κ*B signaling under basal and stress conditions. To explore whether *Prdx6* depletion contributes to NF-*ĸ*B activation, we examined the nuclear translocation of the NF-*ĸ*B/p65 subunit and degradation of its cytoplasmic inhibitor, I-*ĸ*Bα. Immunoblotting of nuclear and cytosolic fractions isolated from Prdx6^+/+^ and *Prdx6^−/−^* mLECs and *Prdx6*-depleted SRA-hLECs revealed that Prdx6 depletion led to I-*ĸ*Bα degradation and increased levels of the phospho-I*ĸ*Bα, facilitating nuclear translocation of NF-*ĸ*B/p65 (Figure 9D,E). Prdx6 overexpression blocked this translocation of the NF-*ĸ*B levels by preventing I-*ĸ*Bα degradation in *Prdx6^−/−^* mLECs, indicating that Prdx6 attenuated NF-*ĸ*B activation (Figure 9F). Taken together, the results revealed that Prdx6 negatively regulates NF-*κ*B signaling under basal and stress conditions.

### 3.10. Redox-Sensitive Transcription Factor NF-κB Was Activated in Prdx6-Deficient and Oxidatively Stressed LECs, Whereas Prdx6 Overexpression Mitigated This Effect

**Figure 10:**
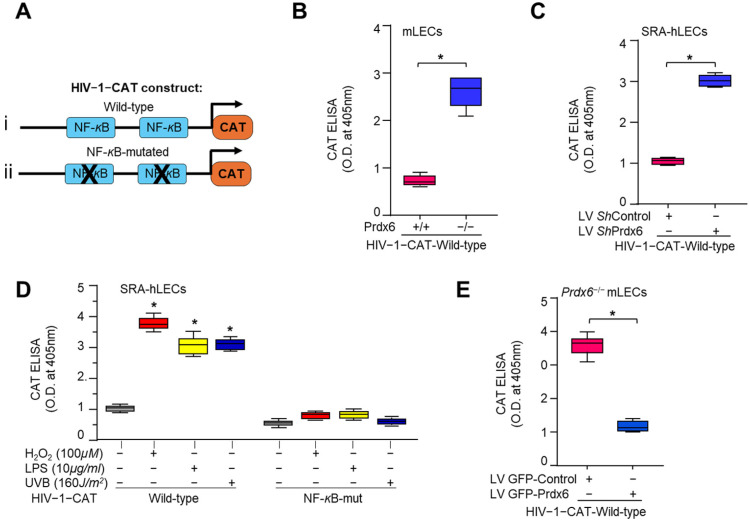
Redox-active transcription factor NF-*ĸ*B was activated in *Prdx6*-deficient LECs and LECs facing oxidative stress but its activity was normalized by Prdx6 overexpression: (**A**) HIV-1 LTR-CAT and its NF-*ĸ*B mutant construct; (**B**,**C**) *Prdx6* deficiency displayed an active form of redox-active transcription factor NF-*ĸ*B. Prdx6^+/+^, *Prdx6^−/−^*, and *Prdx6*-depleted SRA-hLECs were transiently transfected with HIV-1 LTR-CAT; 48 h later, NF-*ĸ*B transcription activity was measured. The data represent the mean ± SD, from four independent repeats (*n* = 4). Prdx6^+/+^ vs. *Prdx6^−/−^* and LV ShControl vs. LV *ShPrdx6*; * *p* < 0.001; (**D**) SRA-hLECs were transfected with HIV-1 LTR-CAT and its NF-*ĸ*B mutant construct; 24 h after post-transfection, these cells were exposed to H_2_O_2_ or LPS or UVB, as indicated in the figure. The transactivation assay was performed after 24 h of oxidative stress exposure and NF-*ĸ*B activity was shown; and (**E**) LV GFP-Control and LV GFP-Prdx6 infected *Prdx6^−/−^* mLECs were transfected with HIV-1 LTR-CAT, and NF-*ĸ*B transcription activity was measured at 48 h. Each value represents the mean ± SD from four independent repeats. Untreated vs. H_2_O_2_/LPS/UVB treatment and LV-GFP-Control vs. LV-GFP-Prdx6; * *p* < 0.001.

Further, we confirmed NF-*κ*B activity by a transactivation assay using an HIV-1 LTR-CAT reporter containing NF-*κ*B binding sites (Figure 10A). NF-*κ*B expression was significantly increased in *Prdx6^−/−^* mLECs or *Prdx6*-depleted SRA-hLECs, as shown in Figure 9. A transactivation assay using an HIV-1 LTR-CAT construct confirmed the activation of NF-*κ*B transcription in *Prdx6*-deficient LECs (Figure 10B,C). Previously, we have shown that CoCl_2_ exposure to HT22 cells significantly increased NF-*κ*B transcriptional activity [123]. HIV-1 LTR-CAT and its mutant (mutated at NF-*κ*B binding sites), and constructed and transfected mLECs were exposed to different oxidants, such as H_2_O_2_, LPS and UVB, and CAT activity was determined. The results demonstrated increased NF-*κ*B transcriptional activity in the HIV-1 LTR-CAT transfected construct in response to oxidative stress, while no activity was observed in the NF-*κ*B sites’ mutated construct (Figure 10D). Prdx6 overexpression in *Prdx6^−/−^* mLECs via LV GFP-Prdx6 transduction significantly attenuated NF-*κ*B transcriptional activity (Figure 10E), confirming the repressive role of Prdx6 on redox-activated NF-*κ*B signaling.

### 3.11. In Silico and Functional Promoter Analyses Revealed That NF-κB Directly Regulates Klf9 Transcription, and This Activity Was Elevated in Aging and Prdx6^−/−^ mLECs

**Figure 11:**
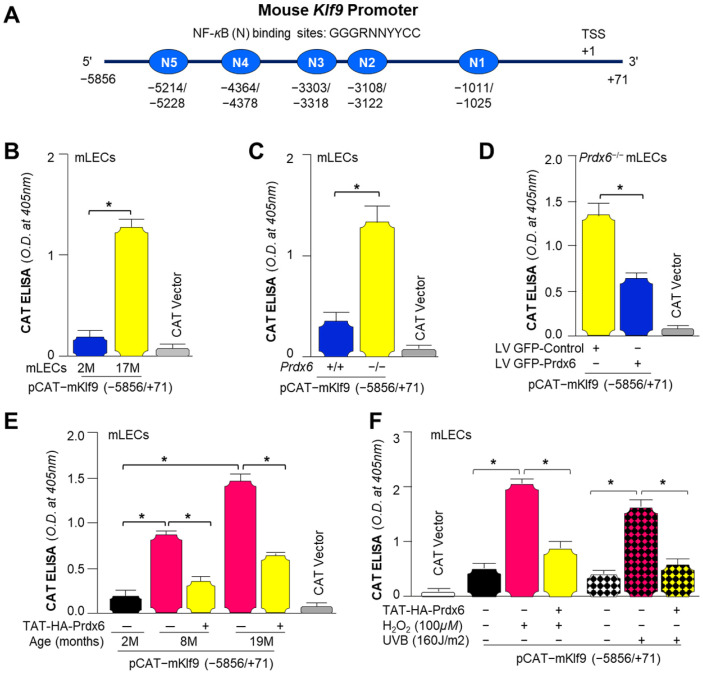
(**A**–**C**) In silico analysis revealed the presence of NF-*ĸ*B binding sites in Klf9 promoter: aged mLECs and *Prdx6^−/−^* mLECs, a model for aging, displayed increased Klf9 promoter activity; (**A**) schematic diagram showing the 5′-construct of mKlf9 promoter, spanning −5856/+71 bps linked to CAT reporter gene, with the position of NF-*ĸ*B binding sequence; (**B**,**C**) mLECs were transiently transfected with the Klf9 promoter, along with the GFP vector. Forty-eight hours post transfection, CAT activity was examined. The data show the mean ± SD from four independent experiments (*n* = 4). 2M vs. 17M-old and Prdx6^+/+^ vs. *Prdx6^−/−^* mLECs; * *p* < 0.001; (**D**) *Prdx6^−/−^* mLECs infected with LV GFP-Control or LV GFP-Prdx6 were transfected with Klf9 promoter, and CAT activity was measured at 48 h. The results represent the mean ± SD from four separate replicates (*n* = 4). LV GFP-Control vs. LV-GFP-Prdx6; * *p* < 0.001; and (**E**,**F**) elevated Klf9 transcriptional activity in aging mLECs or mLECs facing oxidative stress was inhibited by TAT-HA-Prdx6. Aging mLECs and mLECs were transiently transfected with Klf9 promoter construct along with GFP plasmid; 24 h later, aging mLECs were transduced with TAT-HA-Prdx6 (10 µg/mL) for 24 h, and mLECs were transduced with TAT-HA-Prdx6 (10 µg/mL) for 3 h, followed by H_2_O_2_ and/or UVB exposure for 24 h. CAT activity was measured after 24 h. Transfection efficiency was normalized using GFP O.D, measured at Ex485/Em530 nm. The results represent the mean ± SD from four independent experiments (*n* = 4). 2M vs. 8M-old and 19M-old, control vs. TAT-HA-Prdx6, untreated control vs. H_2_O_2_ vs. H_2_O_2_ + TAT-HA-Prdx6, and untreated control vs. UVB vs. UVB + TAT-HA-Prdx6; * *p* < 0.001.

We observed elevated levels of NF-*κ*B and Klf9 in *Prdx6^−/−^* mLECs or aging LECs/LECs facing oxidative stress. Therefore, we were interested to know whether there was any correlation between NF-*κ*B and Klf9. We performed in silico motif discovery analysis using MEME Suites. Bioinformatics analysis identified putative NF-*κ*B binding sites/activating elements in the Klf9 promoter, as shown in Figure 11A. We found increased Klf9 promoter activity in aging mLECs and *Prdx6^−/−^* mLECs (Figure 11B,C), suggesting that Klf9 may be a direct transcriptional target of NF-*κ*B under oxidative stress conditions, as aging mLECs and *Prdx6^−/−^* mLECs bear a higher oxidative load. Overexpression of Prdx6 using LV GFP-Prdx6 in *Prdx6^−/−^* mLECs resulted in marked suppression of Klf9 gene transcription (Figure 11D). Similarly, TAT-HA-Prdx6 delivery inhibited age-associated oxidative stress-induced elevated Klf9 transcription in aging LECs (Figure 11E) as well as oxidant-induced (H_2_O_2_ and UVB) increased Klf9 transcription activity in mLECs (Figure 11F), suggesting that Prdx6 represses NF-*κ*B-mediated Klf9 transcription, particularly under aging and oxidative stress conditions.

### 3.12. Elevated TXNIP Expression and Reduced TRX1 Levels Were Observed in Both Prdx6^−/−^ and Aging mLECs, Implicating a Redox Imbalance Due to Prdx6 Loss

**Figure 12:**
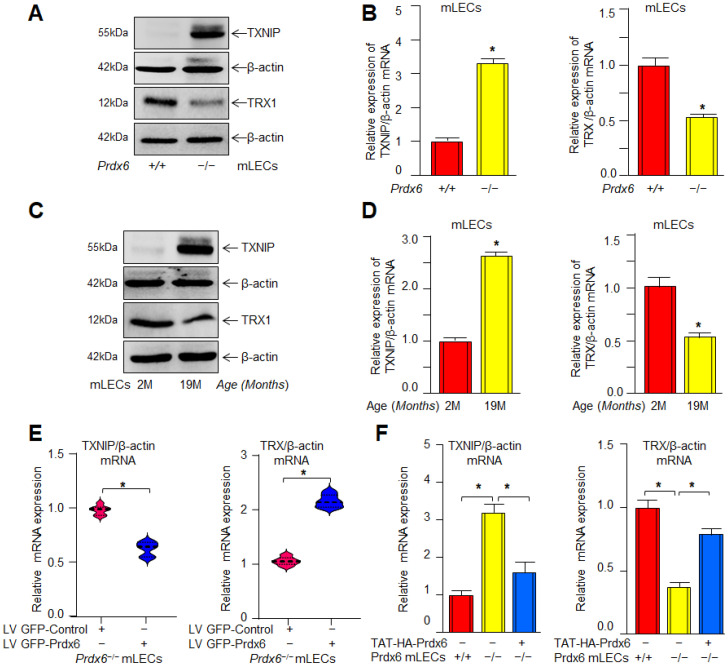
(**A**–**D**) *Prdx6^−/−^* (a model for aging) and aging mLECs showed increased levels of TXNIP with reduced antioxidant gene TRX. Elevated TXNIP levels in *Prdx6^−/−^* and aging mLECs were related to TRX repression. Total protein and RNA were isolated from Prdx6^+/+^, *Prdx6^−/−^* mLECs and aging mLECs and subjected to Western blot and RT-qPCR analysis. Data represent the mean ± SD values of six independent experiments (*n* = 6). Prdx6^+/+^ vs. *Prdx6^−/−^* mLECs samples; young (2-month-old) vs. Old (19-month-old) mLECs samples; * *p* < 0.001; (**E**,**F**) Prdx6 delivery inhibited the elevated TXNIP expression and enhanced the TRX expression in *Prdx6^−/−^* mLECs. Total RNA was isolated from LV GFP-Control and LV GFP-Prdx6 infected *Prdx6^−/−^* mLECs (E) and/or *Prdx6^−/−^* mLECs transduced with TAT-HA-Prdx6 (10 µg/mL) for 24 h. TXNIP and TRX mRNA expression levels were examined by qPCR. The data show the mean ± SD values derived from six separate experiments (*n* = 6). Prdx6^+/+^ vs. *Prdx6^−/−^* mLECs and *Prdx6^−/−^* control vs. *Prdx6^−/−^* with TAT-HA-Prdx6 samples; * *p* < 0.001.

A recent report indicates that TXNIP interacts with Nlrp3 protein and plays an important role in Nlrp3-inflammasome activation by suppressing TRX [97,106,124]. Activated Nlrp3 inflammasome was observed in *Prdx6^−/−^* mLECs, a model for aging as well as aging LECs, as shown in Figure 1, Figure 2 and Figure 3. Therefore, we intended to know the expression level of TXNIP and TRX in aging LECs and *Prdx6^−/−^* mLECs. Protein and mRNA expression analyses showed a significant increase in TXNIP, with a decrease in TRX expression in *Prdx6*-deficient (*Prdx6^−/−^*) in comparison to Prdx6 wild-type (Prdx6^+/+^) mLECs (Figure 12A,B), and in aged (19-month-old) vs. young (2-month-old) mLECs (Figure 12C,D). Prdx6 overexpression by LV GFP-Prdx6 (Figure 12E) or delivery by TAT-HA-Prdx6 transduction (Figure 12F) markedly reversed the decreased expression of TXNIP with increased TRX expression in *Prdx6^−/−^* mLECs. Data demonstrated that Prdx6 modulates redox balance by regulating the TXNIP–TRX system, facilitating protective cellular microenvironment against age and oxidative stress-associated with oxidative-induced Nlrp3 inflammasome-mediated inflammatory signaling.

## 4. Discussion

Aging is a normal physiological process and is found to be linked to the overaccumulation of oxidative load-induced different types of death pathways, like apoptosis, pyroptosis, ferroptosis, penoptosis and necroptosis, and thereby contributes to various age-related diseases [50,125,126,127,128,129]. Previously, we have shown that during aging and oxidative stress conditions, antioxidant Prdx6 expression and activities decline, which is directly associated with age-related disorders, including cataractogenesis and glaucoma [22,60,62,114]. 1-Cys Prdx6 is a multifunctional antioxidant enzyme with GPx, PLA_2_, and LPCAT activities. Prdx6 uses glutathione as an electron donor while other members of peroxiredoxins utilize thioredoxin as a physiological reductant [36]. Redox-active *Prdx6*-deficient mLECs or aging LECs show increased expression and activation of Nlrp3 inflammasome and its inflammatory components with ROS production [60]. Furthermore, the exposure of extrinsic and intrinsic oxidative stressors, like UVB, H_2_O_2_, LPS, tunicamycin, glutamate, cobalt chloride, paraquat, etc., to the cells leads to increased ROS generation with a reduction in antioxidant genes, including Prdx6 [20,51,58,63,64,117,120,123,130,131]. It has been shown that aging cells, having a low abundance of Prdx6 as well as *Prdx6-*deficient cells, are more vulnerable to oxidative stress-induced inflammatory or noninflammatory cell death [20,22,51,58,61,62,121,123,131]. In the present study, Prdx6 knockdown human/mouse LECs displayed increased levels of Nlrp3 inflammasome and its inflammatory component expression and showed higher sensitivity to oxidants such as H_2_O_2_, LPS, and UVB-induced oxidative cell death (Figure 1, Figure 2 and Figure 3). It has been reported that Prdx6 silencing enhanced the intracellular ROS level and sensitized senescent cells or normal cells to H_2_O_2_-induced cell death [22,62,114,121,132]. H_2_O_2_-exposed ARPE-19 cells showed increased ROS production with reduced cell viability and antioxidants [45]. Moreover, it has been demonstrated that epithelial cells play a crucial role in detecting danger signals and initiating the immune response to protect the cells and tissue integrity [60,133]. H_2_O_2_ serves a dual role, acting both as a priming and activation signal for the Nlrp3 inflammasome, which leads to the release of bioactive cytokines, such as IL-1β, by activating Caspase-1 [134]. It has been reported that H_2_O_2_ exposure remarkably increased the expression and release of TNFα, IL-1β, and IL-6, and the maturation of IL-1β, and that the event was Nlrp3 inflammasome-dependent in tenocytes [135]. Ultraviolet (UV) radiation from sunlight directly affects skin and eye, leading to damage to the intracellular DNA molecules; UVB-induced DNA damage triggers Nlrp3 inflammasome activation [121,136]. The UVB exposure dose dependently increase the Nlrp3 and IL-1β mRNA expression in human keratinocytes [136]. These findings are in agreement with our findings (Figure 2 and Figure 7). UVB and LPS exposure results in mitochondrial damage, ROS production, and LDH release, which further leads to the enhanced expression of Nlrp3, ASC oligomerization, and the activation of Caspase-1. This cascade subsequently facilitates the release of bioactive IL-1β and IL-18, and GSDMD-mediated responses [137]. UVB exposure activated the Nlrp3 inflammasome and promoted the formation of ASC specks and enhanced the activity of Caspase-1, triggering the secretion of IL-1β, IL-18, and LDH in human corneal epithelial (HCE) cells [138]. Oxidative stress induced by UV radiation activates the NF-*ĸ*B, which leads to an increase in Nlrp3 inflammatory components and their pro-inflammatory components. Studies have shown that a polynucleotide and polynucleotide mixture (containing antioxidants such as glutathione and hyaluronic acid) reduced melanogenesis in UVB-irradiated keratinocytes and animal skin by lowering oxidative stress and suppressing NF-*ĸ*B and Nlrp3 inflammasome activation [139], suggesting a prime role of antioxidant molecules. In our study, we found that aging hLECs or *Prdx6^−/−^* mLECs or Prdx6 knockdown mLECs had elevated levels of redox-sensitive transcription factors, NF-*κ*B and Klf9, along with Nlrp3 inflammasome and inflammatory components (Figure 8, Figure 10 and Figure 11). Interestingly, *Prdx6*-deficient mice displayed lens opacity and LECs were more vulnerable to oxidative stress and showed phenotypic changes [58]. However, several recent studies reveal that the oxidative stress-induced Nlrp3 inflammasome-mediated inflammatory pathway is one of the causative factors in the development of cataracts [113,140,141,142,143,144,145,146,147,148].

Moreover, antioxidant delivery, or enhancing the antioxidant gene(s) expression via activating the transcription factors by means of phytochemicals or FDA-approved drugs, protects the cells against oxidative damage [19,20,22,60,62,66,114]. Prdx6 delivery by TAT-HA-Prdx6 protein or by overexpression inhibits the age-associated inflammasome activation by normalizing the ROS production, inhibits the Nlrp3 and its downstream target genes, and thereby protects the cells against UVB or H_2_O_2_-induced cell death (Figure 4, Figure 5, Figure 6 and Figure 7). Prdx6 overexpression protects RPE cells (ARPE-19) from oxidative injury by normalizing ROS generation [45]. Glaucomatous TM cells displayed a lower level of Prdx6 with increased expression of ECM proteins and senescence markers, and increased ROS and bioactive TGFβ levels. Prdx6 delivery in glaucomatous cells attenuated the senescence process by reducing DNA damage, ROS and TGFβ levels, and by reducing the expression of ECM proteins and senescence markers [131]. *Prdx6^−/−^* LECs showed reduced LEDGF, HSP27, and αB-crystallin transcription and abnormal cell phenotype, which was restored by Prdx6 overexpression [58]. In a previous study, we have shown that an optimum dose of sulforaphane (SFN) enhanced the Prdx6 expression via Nrf2 activation and protected the cells against oxidative damage [20,61]. SFN alleviated Nlrp3 inflammasome-related proteins and cytokines and reduced the ROS generation induced by LPS and Nlrp3 agonists in RAW264.7 cells [149]. The FDA-approved anti-diabetic drug, Metformin, has anti-aging properties and enhances antioxidant gene expression by activating the AMP-activated protein kinase (AMPK)/Bmal1/Nrf2 antioxidant axis, thereby protecting cells. We have recently shown that Metformin treatment of LECs protects the cells against oxidative stress by regulating the antioxidant defense system through activation of the AMPK/Bmal1/Nrf2 Axis [22]. Activated Nlrp3 inflammasome and the increased expression of its target protein Caspase-1, IL-1β, GSDMD, as well as mTOR with reduced expression of pAMPK, was observed in diabetic cardiomyopathy (DCM). Metformin treatment activates AMPK/autophagy and subsequently inhibits the Nlrp3 inflammasome in DCM, indicating that Metformin has cardioprotective and anti-inflammatory properties [150]. In this scenario, we think that activation of the naturally occurring antioxidant system by means of Metformin application or extrinsic supply of Prdx6 to cells and tissues should be a vital approach to block oxidative Nlrp3-induced pathological signaling. LPS is known as an inflammasome inducer, which activates NF-*ĸ*B by inducing phosphorylation of I*ĸ*Bα and degradation of the I*ĸ*Bα. It is also known that LPS or nigericin-induced Nlrp3 inflammasome activation increases the production of IL-1β, ASC speck formation, ASC oligomerization, and mitochondrial ROS generation, and that the process was inhibited by vitamin B6 [151]. Thus, in this study, we have used a standard molecule to induce the Nlrp3 inflammasome pathway to validate our results.

*Prdx6-*depleted LECs and LECs facing oxidative stress demonstrated the increased Nlrp3 transcription that is restored via Prdx6 delivery (Figure 8). In our previous study, we have shown the elevated Nlrp3 transcription in *Prdx6^−/−^* mLECs and/or mLECs facing H_2_O_2_-induced oxidative stress [60]. Angiotensin II and LPS treatments significantly enhanced the Nlrp3 promoter activity in the human renal tubular epithelial (RTE) cell line and HK-2 cells [152]. We observed increased NF-*ĸ*B mRNA expression and nuclear NF-*ĸ*B with I*ĸ*B degradation and elevated pI*ĸ*B in cytosol in *Prdx6*-deficient LECs (Figure 9). Activated NF-*ĸ*B transcription was observed in *Prdx6*-deficient LECs and LECs facing oxidative stress (Figure 9). In this study, bioinformatics analysis revealed the presence of NF-*ĸ*B and Klf9 binding sites in the Nlrp3 gene promoter (Figure 10A). LPS treatment significantly enhanced the NF-*ĸ*B transcription and nuclear NF-*ĸ*B levels in Raw264.7 cells. LPS administered to adult male Sprague Dawley rats showed markedly enhanced Nlrp3, Caspase-1, IL-1β, IL-18, and NF-*ĸ*B activity and expression [153]. NF-*ĸ*B/p65 directly binds to the Nlrp3 promoter and modulates its transcription. Similarly, our study revealed that NF-*κ*B is a regulator of Nlrp3 transcription in our model system in response to stressors. Furthermore, we observed elevated Klf9 expression in *Prdx6*-deficient cells as well as in aging cells. We and others have shown that the overaccumulation of Klf9 repressed the Prdx6 expression, leading to increased ROS generation. Klf9 overexpressed cells are more susceptible to oxidative stress, while Klf9 knockdown cells are resistant to oxidative damage [20,22,60]. In COPD mouse models, Klf9 expression was found to be elevated, while depletion reduced the Nlrp3 inflammasome-driven airway inflammation through the miR-494-3p/PTEN axis. Klf9 binds to the miR-494-3p promoter, suppresses PTEN expression, and thereby enhances Nlrp3 inflammasome-mediated inflammatory responses. These findings highlighted Klf9, miR-494-3p, and PTEN as potential diagnostic and therapeutic targets for COPD [93]. We observed that *Prdx6^−/−^*, aging LECs, or LECs facing various kinds of oxidative stress showed increased Klf9 promoter activity, which was normalized with the delivery of Prdx6. Interestingly, bioinformatic analysis revealed the presence of multiple functional NF-*ĸ*B sites in the Klf9 gene promoter (Figure 11), suggesting that Klf9 is regulated by NF-*κ*B during the redox cellular microenvironment. TBHQ protects against bronchopulmonary dysplasia (BPD) in hyperoxic mice by enhancing Nrf2-mediated antioxidant defense, suppressing Nlrp3 inflammasome activation and pyroptosis, and promoting alveolar maturation. Thus, Nrf2 activation and Nlrp3 inhibition represent potential therapeutic strategies for BPD [154]. Furthermore, our data revealed that *Prdx6^−/−^* mLECs, a model for aging or aging cells, had increased expression of TXNIP, which was directly associated with the reduced expression of TRX (Figure 12). TXNIP inhibits biological function by negatively regulating the bioavailability of TRX in hLECs, which is an important protein that controls oxidative damage in almost all eukaryotic cells [155]. Nitrosative and oxidative stress-induced nuclear translocation of TRX1 is linked to its intracellular compartmentalization and activation of the ERK1/2 MAP kinases, which play a critical role in the negative regulation of TXNIP gene expression [156]. Surprisingly, Prdx6 overexpression or TAT-HA-Prdx6 delivery to *Prdx6^−/−^* mLECs suppressed the elevated TXNIP expression, thereby enhancing TRX expression (Figure 12). During oxidative conditions, TRX expression declines with an increase in TXNIP levels. Elevated TXNIP led to activation of the Nlrp3 inflammasome by interacting with Nlrp3, leading to pyroptotic cell death [63,144,157]. TRX1 knockdown aggravated the Nlrp3 activation, while overexpression remarkably inhibited the Nlrp3 activation. TRX1 overexpression inhibited TXNIP and Nlrp3-mediated pyroptosis by weakening the interaction between TXNIP and Nlrp3 in vivo and in vitro in Alzheimer’s disease (AD) [158].

## 5. Conclusions

**Figure 13:**
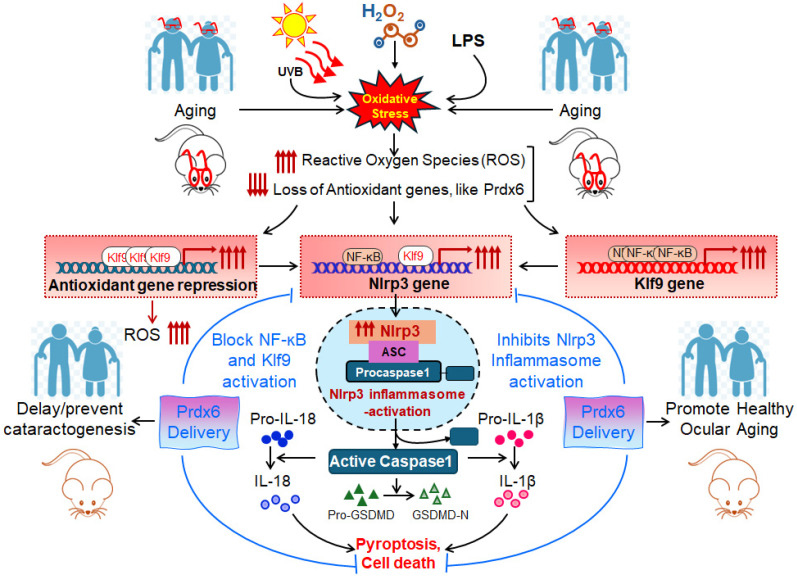
Prdx6’s role in the protection and survival of lens epithelial cells in aging and oxidant-induced oxidative stress. During aging and oxidative stress, the loss of antioxidant defense in LECs leads to excessive ROS accumulation, triggering Nlrp3 inflammasome activation through NF-*κ*B and Klf9 signaling. Activated Klf9 further amplifies oxidative stress by increasing ROS levels and suppressing antioxidant gene expression, while NF-*κ*B directly enhances Klf9 transcription, together promoting sustained inflammasome activation. Aberrantly activated Nlrp3 assembles with ASC and proCaspase-1, resulting in the activation of Caspase-1, maturation of IL-1β/IL-18, cleavage of GSDMD, and induction of pyroptotic cell death. The delivery of Prdx6 suppresses NF-*κ*B and Klf9 activation, reduces ROS accumulation, restores antioxidant defense, and inhibits Nlrp3 inflammasome activation, thereby promoting healthy ocular aging and delaying cataract formation.

Prdx6 plays a critical role in protecting LECs against inflammation induced by aging and oxidative stress. During aging, a progressive loss of antioxidant defense leads to an excessive accumulation of ROS, which triggers activation of the Nlrp3 inflammasome through NF-*κ*B and Klf9 signaling pathways. Our data demonstrated that *Prdx6* deficiency in LECs under aging or oxidative stress conditions drives aberrant activation of the Nlrp3 inflammasome-mediated inflammatory pathway. This activation consists in the recruitment of the adaptor protein ASC, which in turn recruits pro-Caspase-1 to assemble the Nlrp3–ASC–proCaspase-1 inflammasome complex. Activated Caspase-1 cleaves the pro-inflammatory cytokines, pro-IL-1β and pro-IL-18, into their mature, biologically active forms, IL-1β and IL-18. Caspase-1 also promotes the activation of GSDMD, leading to pore formation and the release of bioactive cytokines, ultimately causing the pyroptotic cell death. Interestingly, the delivery of Prdx6 suppressed the oxidative-induced aberrant activation of Nlrp3 inflammasome-mediated inflammatory cell death. Further, the elevated Nlrp3 transactivation observed in *Prdx6*-depleted LECs or LECs facing oxidative stress was inhibited by Prdx6 delivery. As shown in Figure 8 and Figure 11, the Nlrp3 promoter bears NF-*ĸ*B and Klf9 binding sites; NF-*ĸ*B binding sites are present in the Klf9 promoter. *Prdx6^−/−^* mLECs or Prdx6-depleted SRA-hLECs showed elevated nuclear NF-*ĸ*B and increased its transcription. Elevated nuclear NF-*ĸ*B and its increased transcription, in response to aging or oxidative stress, were downregulated by the addition of Prdx6. Aging LECs or LECs facing oxidative stress showed increased expression and activation of Klf9. TXNIP expression is needed for Nlrp3 activation; we found the increased expression of TXNIP with reduced expression of TRX in *Prdx6^−/−^* and aging LECs. This aberrant process could be reversed by the supply of Prdx6. These data indicate the involvement of NF-*ĸ*B/Klf9 and TXNIP in the abnormal Nlrp3 inflammasome regulation in response to oxidative stress and aging and indicate that the abnormal/pathological pathway can be abated via the supply of an antioxidant, Prdx6 (Figure 13).

## Figures and Tables

**Table 1:** RT-qPCR primer sequences.

Gene	Forward Primer (5′ to 3′)	Reverse Primer (5′ to 3′)	Gene Accession Number
*mNlrp3*	TCACAACTCGCCCAAGGAGGAA	AAGAGACCACGGCAGAAGCTAG	NM_145827
*mASC*	CTGCTCAGAGTACAGCCAGAAC	CTGTCCTTCAGTCAGCACACTG	NM_023258
*mCas-1*	GGCACATTTCCAGGACTGACTG	GCAAGACGTGTACGAGTGGTTG	NM_009807
*mIL-1β*	TGGACCTTCCAGGATGAGGACA	GTTCATCTCGGAGCCTGTAGTG	NM_008361
*mIL-18*	GACAGCCTGTGTTCGAGGATATG	TGTTCTTACAGGAGAGGGTAGAC	NM_008360
*mGSDMD*	GGTGCTTGACTCTGGAGAACTG	GCTGCTTTGACAGCACCGTTGT	NM_026960
*mPrdx6*	ATCCGCTTCCACGATTTCCTGG	TTCCTCTTGGCAAACTCTGGCG	NM_007453
*mTXNIP*	ATGGCCAGACCAAAGTGTTC	GGCTGTCTTGAGAGTCGTCC	Wang C. et al., 2019 [100]
*mTRX*	CAACAGCCAAAATGGTGAAGCTG	AGGTTTTAAACAGCTG	Wang C. et al., 2019 [100]
*mNF-ĸB*	TCCTGTTCGAGTCTCCATGCAG	GGTCTCATAGGTCCTTTTGCGC	NM_009045
*mβ-actin*	CTAAGGCCAACCGTGAAAAG	ACCAGAGGCATACAGGGACA	NM_007393.3
*hNlrp3*	GGACTGAAGCACCTGTTGTGCA	TCCTGAGTCTCCCAAGGCATTC	NM_004895.1
*hASC*	AGCTCACCGCTAACGTGCTGC	GCTTGGCTGCCGACTGAGGAG	NM_013258
*hCas-1*	GCTGAGGTTGACATCACAGGCA	TGCTGTCAGAGGTCTTGTGCTC	NM_033292
*hIL-1β*	CCACAGACCTTCCAGGAGAATG	CCTTGATGTTATCAGGAGGATTCA	NM_000576
*hIL-18*	GATAGCCAGCCTAGAGGTATGG	GATAGCCAGCCTAGAGGTATGG	NM_001562
*hGSDMD*	ATGAGGTGCCTCCACAACTTCC	CCAGTTCCTTGGAGATGGTCTC	NM_024736
*hPrdx6*	TCAATAGACAGTGTTGAGGACCA	TTTCTGTGGGCTCTTCACAA	NM_004905.2
*hNF-ĸB*	TGAACCGAAACTCTGGCAGCTG	CATCAGCTTGCGAAAAGGAGCC	NM_021975
*hβ-actin*	CCAACCGCGAGAAGATGA	CCAGAGGCGTACAGGGATAG	NM_001101.3

## Data Availability

The original contributions presented in this study are included in the article. Further inquiries can be directed to the corresponding authors.

## Abbreviations

The abbreviations used throughout this manuscript are as follows:

Nlrp3	Nod-Like Receptor Pyrin 3
Prdx6	Peroxiredoxin 6
ROS	Reactive Oxygen Species
Klf9	Kruppel-like factor 9
LECs	Lens Epithelial Cells
Prdx6^−/−^	*Prdx6*-deficient
ARDs	Age-related diseases
Prdxs	Peroxiredoxin
GSH	Glutathione
GPx	GSH peroxidase
aiPLA2	Ca^2+^ -independent phospholipase A2
LPCAT	Lysophosphatidylcholine acyltransferase
ER	Endoplasmic Reticulum
MMP9	Matrix metalloproteinase 9
PE	Preeclampsia
COPD	Chronic obstructive pulmonary disease
TXNIP	Thioredoxin-interacting protein
TRX	Thioredoxin
hLECs	Human lens epithelial cells
mLECs	Mouse lens epithelial cells
LV	Lentiviral
RIPA	Radioimmunoprecipitation assay
H2-DCF-DA	Fluorescent dye 2′,7′-dichlorofluorescin diacetate
DCF	Dichlorofluorescein
HBSS	Hank’s Balanced Salt Solution
IL-1β	Interleukin-1 beta
IL-18	Interleukin-18
O.D.	Optical Density
PMSF	Phenylmethylsulfonyl fluoride
NF-*κ*B	Nuclear factor kappa B
CAT	Chloramphenicol Acetyltransferase (CAT)
GSDMD	Gasdermin D
H_2_O_2_	Hydrogen Peroxide
UVB	Ultraviolet B
